# The C-Terminal Domain of Nrf1 Negatively Regulates the Full-Length CNC-bZIP Factor and Its Shorter Isoform LCR-F1/Nrf1β; Both Are Also Inhibited by the Small Dominant-Negative Nrf1γ/δ Isoforms that Down-Regulate ARE-Battery Gene Expression

**DOI:** 10.1371/journal.pone.0109159

**Published:** 2014-10-07

**Authors:** Yiguo Zhang, Lu Qiu, Shaojun Li, Yuancai Xiang, Jiayu Chen, Yonggang Ren

**Affiliations:** The Laboratory of Cell Biochemistry and Gene Regulation, College of Medical Bioengineering and Faculty of Life Sciences, Chongqing University, Shapingba District, Chongqing, China; North Carolina State University, United States of America

## Abstract

The C-terminal domain (CTD, aa 686–741) of nuclear factor-erythroid 2 p45-related factor 1 (Nrf1) shares 53% amino acid sequence identity with the equivalent Neh3 domain of Nrf2, a homologous transcription factor. The Neh3 positively regulates Nrf2, but whether the Neh3-like (Neh3L) CTD of Nrf1 has a similar role in regulating Nrf1-target gene expression is unknown. Herein, we report that CTD negatively regulates the full-length Nrf1 (i.e. 120-kDa glycoprotein and 95-kDa deglycoprotein) and its shorter isoform LCR-F1/Nrf1β (55-kDa). Attachment of its CTD-adjoining 112-aa to the C-terminus of Nrf2 yields the chimaeric Nrf2-C112^Nrf1^ factor with a markedly decreased activity. Live-cell imaging of GFP-CTD reveals that the extra-nuclear portion of the fusion protein is allowed to associate with the endoplasmic reticulum (ER) membrane through the amphipathic Neh3L region of Nrf1 and its basic c-tail. Thus removal of either the entire CTD or the essential Neh3L portion within CTD from Nrf1, LCR-F1/Nrf1β and Nrf2-C112^Nrf1^, results in an increase in their transcriptional ability to regulate antioxidant response element (ARE)-driven reporter genes. Further examinations unravel that two smaller isoforms, 36-kDa Nrf1γ and 25-kDa Nrf1δ, act as dominant-negative inhibitors to compete against Nrf1, LCR-F1/Nrf1β and Nrf2. Relative to Nrf1, LCR-F1/Nrf1β is a weak activator, that is positively regulated by its Asn/Ser/Thr-rich (NST) domain and acidic domain 2 (AD2). Like AD1 of Nrf1, both AD2 and NST domain of LCR-F1/Nrf1β fused within two different chimaeric contexts to yield Gal4D:Nrf1β^607^ and Nrf1β:C270^Nrf2^, positively regulate their transactivation activity of cognate Gal4- and Nrf2-target reporter genes. More importantly, differential expression of endogenous ARE-battery genes is attributable to up-regulation by Nrf1 and LCR-F1/Nrf1β and down-regulation by Nrf1γ and Nrf1δ.

## Introduction

Collectively, the cap’n’collar (CNC) family of transcription factors controls a variety of critical homeostatic and developmental pathways through regulating the expression of antioxidant response element (ARE)-driven genes, encoding antioxidant proteins, detoxification enzymes, metabolic enzymes and 26S proteosomal subunits [1], [2]. The CNC family comprises the founding member *Drosophila* Cnc protein, the *Caenorhabditis elegans* protein skinhead-1 (Skn-1), the vertebrate activator nuclear factor-erythroid 2 (NF-E2) p45 subunit, NF-E2-related factor 1 (Nrf1, including its long form transcription factor 11 (TCF11) and its short form Locus control region-factor 1 (LCR-F1, also called Nrf1β)), Nrf2 and Nrf3, as well as the transcription repressors Bach1 (BTB and CNC homolog 1) and Bach2 [1], [3]–[6]. All other family members except Skn-1 form a functional heterodimer with a small Maf factor or other basic region-leucine zipper (bZIP) proteins, before binding to ARE sequences in their target gene promoters [7]–[9].

Amongst mammalian CNC-bZIP proteins, Nrf1 and Nrf2 are two principal factors to regulate ARE-driven cytoprotective genes against cellular stress [10]–[12]. Surprisingly, most of researches have focused on Nrf2, that is considered to be a master regulator of adaptive responses to oxidative stressors and electrophiles [13], [14]. However, Nrf2 is dispensable for development because global knockout of its gene in mice yields viable animals [15]. Specifically, *Nrf2* ^−/−^ mice do not develop any spontaneous cancer, although they are more susceptible than wild-type mice to carcinogens [16]. By contrast, relatively less is known about Nrf1, although global knockout of the LCR-F1/Nrf1β-encoding sequence from its gene locus in the mouse causes embryonic lethality and severe oxidative stress [17]–[20]. Moreover, conditional knockout of *Nrf1* (also called *nfe2l1*) in the liver, brain and bone results in non-alcoholic steatohepatitis (NASH) and hepatoma [21], [22], neurodegeneration [23], [24], and reduced bone size [25]. These studies demonstrate that Nrf1 fulfils a unique and indispensable function responsible for maintaining cellular homeostasis and organ integrity.

**Figure 1:**
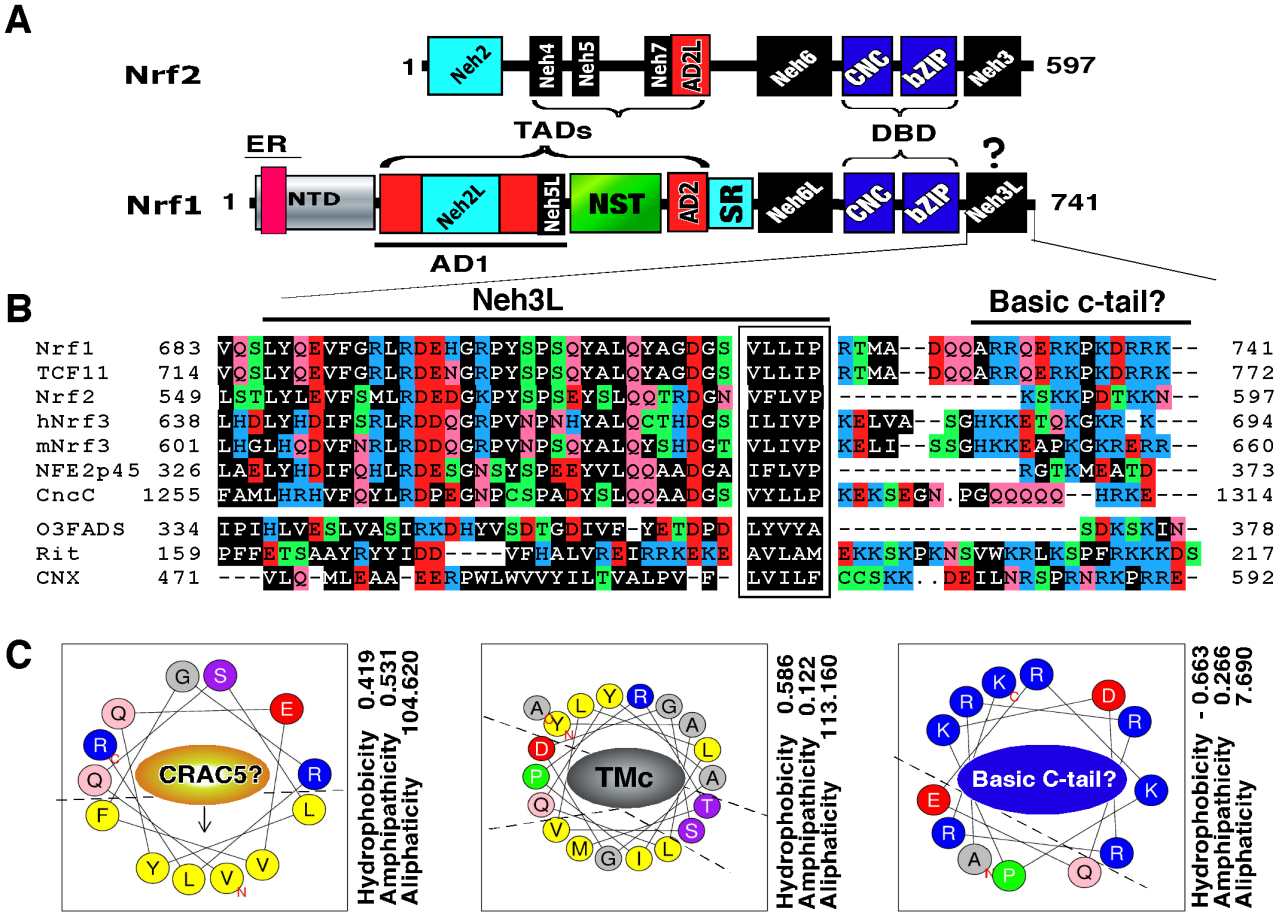
The Neh3L-containing CTD of Nrf1 is conserved in the CNC-bZIP family. (**A**) Schematic representation of discrete domains of Nrf1 and Nrf2. Locations of the ER signal, transactivation domains (TADs, including AD1, NST and AD2), DNA-binding domain (DBD, including CNC and bZIP) are indicated within Nrf1. The Neh3L region is situated within the C-terminal domain (CTD) of Nrf1. The positive regulation of Nrf2 by its Neh3 domain occurs through direct interaction with CHD6 [37], but it is not identified as one of Nrf1-interacting proteins [71]. (**B**) An alignment of amino acids covering CTD in Nrf1 and other CNC-bZIP factors with ER-resident proteins. The CNC family comprises both water-soluble members (i.e. NF-E2p45 and Nrf2) and membrane-bound NHB1-CNC members (including Nrf1, TCF11, Nrf3, CncC, and Skn-1, albeit the latter lacks both the corresponding ZIP and Neh3L regions). The distinction between Neh3L and Neh3 from Nrf1 and Nrf2 is attributable to different positioning relatively to membranes. Amongst the NHB1-CNC proteins, the core Neh3L is conserved with an ER-resident protein, omeg-3 fatty acid desaturase (O3FADS). Its N-terminally flanking CRAC motif is present in Nrf1 (numbered as CRAC5), TCF11 and Nrf3, but is absent from other members. The C-terminal basic cluster is predicted to possess an ER-retention signal (K/RxK/R), which ensembles to those in calnexin (CNX), O3FADS and Rit (Ras-like protein in all tissues). The conversed hydrophobic pentapeptide is boxed due to the representative in Nrf2 that is essential for its interaction with CHD6 [37]. (**C**) Bioinformatic prediction of three discrete regions within CTD of Nrf1. It is proposed that both CRAC5 and TMc sequences could be wheeled into two relative stable amphipathic helices only upon interaction with amphipathic membranes, whilst a positively-charged helix folded by the basic C-terminal peptide could interact electrically with the putative negatively-charged head group of membrane lipids. Three physico-chemical parameters related with the helical folding (i.e. aliphaticity, hydropathicity and amphipathicity) were calculated using the ProParam tool (http://web.expasy.org/protparam/).

It is axiomatic that the distinct biological functions of Nrf1 and Nrf2 are determined by differences in their primary structures. It is clearly reported that the function of Nrf2 is dictated by its seven structural domains, namely Nrf2-ECH homology 1 (Neh1) to Neh7 (Fig. 1A), which are conserved amongst different metazoan species [26], [27]. The central Neh1 domain comprises both the CNC and bZIP regions, which mediate its DNA-binding to ARE and heterodimerization with other bZIP proteins [8], [9], [28], [29]. The N-terminal Neh2 domain contains a redox-sensitive Kelch-like ECH-associated protein 1 (Keap1)-binding degron, that targets the normal homeostatic Nrf2 protein to the ubiquitin ligase cullin 3/Rbx1-dependent proteasomal degradation pathway [30]–[32]; a two-site substrate recognition model was proposed that the forked-stem homodimer of Keap1 binds to the DLG and ETGE motifs within this domain [33]–[36]. The C-terminal Neh3 domain is required for Nrf2 activity through interaction with chromo-ATPase/helicase DNA-binding protein 6 (CHD6) [37]. The central Neh4 and Neh5 are two transactivation domains (TADs) that contribute to positive regulation of Nrf2 by recruiting CRE-binding protein (CREB)-binding protein (CBP) [38], [39]; the activity is controlled through a tight interaction with nuclear receptor-associated coactivator 3 (RAC3, also called steroid receptor coactivator-3 (SRC-3)) [40]. The Neh6 domain, adjacent to the N-terminus of the Neh1 domain, acts as a redox-insensitive β-transducin repeat-containing protein (β-TrCP)-binding degron that negatively regulates Nrf2 [41], [42]. The newly-defined Neh7 domain can directly bind to retinoic X receptor α (RXRα), allowing inhibition of Nrf2 activity [27]. In the light of the above information, similar but different structural domains of Nrf1 are identified on the basis of the amino acid sequence similarity with other CNC-bZIP proteins [43].

Significantly, the unique biological function of Nrf1 is dictated by its specific biophysio-chemical properties, such as those provided by the N-terminal domain (NTD, that negatively regulates this CNC-bZIP factor) and the central Asn/Ser/Thr-rich (NST) glycodomain, which are absent from Nrf2. Our previous work showed that negative regulation of Nrf1 by its NTD is associated with the endoplasmic reticulum (ER) through its N-terminal homology box 1 (NHB1) signal sequence, which lacks a signal peptidase cleavage site [43], [44]. Upon translation of Nrf1, its NHB1 sequence enables the newly-synthesized polypeptide to be integrated in a specific topological orientation within and around the membranes [45], [46]. During topological folding of Nrf1, it is anchored within the ER through its NHB1-associated transmembrane-1 (TM1) region. The connecting TAD sequences, that comprise acidic domain 1 (AD1), NST glycodomain, AD2 and possibly serine-repeat (SR) domain, are transiently translocated into the ER lumen. The NST domain is therein glycosylated to allow Nrf1 to represent an inactive 120-kDa glycoprotein, because the luminal TAD is unlikely to transactivate the nuclear target genes, although its DNA-binding CNC-bZIP domain is positioned on the cyto/nucloplasmic side of membranes. When stimulated by biological cues, the TAD sequences are partially partitioned out of the ER and retrotranslocated across membranes into the cyto/nucloplasmic compartments, where Nrf1 is deglycosylated to yield an active 95-kDa factor because its TADs can gain access to the general transcriptional machinery, enabling transactivation of its target genes. Therefore, the membrane-topological organization of Nrf1 dictates selective post-translational processing of this CNC-bZIP protein to generate a cleaved activator (i.e. 85-kDa), a short weak activator (e.g. 55-kDa LCR-F1/Nrf1β), and other small dominant-negative isoforms (e.g. 36-kDa Nrf1γ and 25-kDa Nrf1δ). However, it is not clear how these Nrf1 isoforms together control the overall transcriptional ability to fine-tune target gene expression to an extent to which is required for cellular homeostasis and organ integrity.

Within AD1, the Neh2-like (Neh2L) subdomain (aa 156–242) does not negatively regulate Nrf1 *via* a Keap1-dependent ubiquitin proteasomal degradation pathway [3], [43], although the interaction was examined in total cell lysates [47]. Conversely, Neh2L contributes to the stability of Nrf1, particularly the 120-kDa glycoprotein [48]. The Neh4-like (Neh4L) subdomain is lost in Nrf1 because it is removed by alternative splicing of the transcript encoding the longer isoform TCF11 [49], [50]. The Neh5-like (Neh5L) subdomain (aa 280–298) is essential for AD1-mediated transactivation by Nrf1 [12], [45]. Similarly, AD2 (and possibly SR domain) appears to positively regulate Nrf1, but its contribution to the short LCR-F1/Nrf1β is unclear. By contrast, the Neh6-like (Neh6L) domain (situated between the TADs and DNA-binding domain) negatively regulate Nrf1 through the β-TrCP-mediated and/or PEST (Pro/Glu/Ser/Thr-enriched degron)-dependent proteolytic degradation pathways [48], [51], but its negative effect exerted on LCR-F1/Nrf1β is unknown. Lastly, the C-terminal domain (CTD, aa 686–741) of Nrf1 comprises the major Neh3-like (Neh3L, including a CRAC5 motif and TMc) region and a basic c-tail (BCT) (Fig. 1, B and C), but whether CTD has an effect on Nrf1 similar to the Neh3 domain in Nrf2 is not elucidated.

In the present study we have examined whether: (i) the CTD of Nrf1 exerts a positive or negative effect on its ability to regulate ARE-battery genes; (ii) attachment of the CTD to Nrf2 alters the transactivation activity of the resulting chimaeric factor; (iii) the 36-kDa Nrf1γ or 25-kDa Nrf1δ (both lacking all TAD regions) act as a *bona fide* dominant-negative form that competitively inhibits wild-type Nrf1 and Nrf2; (iv) the 55-kDa Nrf1β acts as a weak activator and is positively regulated by its AD2 and NST domains; and (v) expression of endogenous ARE-driven genes is differentially regulated by distinct Nrf1 isoforms.

## Experimental

### Chemicals and antibodies

All chemicals were of the highest quality commercially available. Proteinase K (PK) was obtained from New England Biolabs. Mouse monoclonal antibodies against the epitope V5 and Xpress were from Invitrogen Ltd. Antisera against Nrf1 or Nrf2 were produced in rabbits by commercial companies.

### Expression constructs

Expression constructs for full-length mouse Nrf1, Nrf1β, Nrf1γ, Nrf1δ, Nrf2 and its mutants, along with a variety of other expression constructs for GFP-CTD fusion protein, and their mutants lacking various lengths of CTD in Nrf1, have been created as described previously [12], [43]. A series of expression constructs for Gal4D-Nrf1 and Gal4D-Nrf1 were generated by ligating cDNA fragments that encode various lengths of Nrf1 to the 3′-end of cDNA sequence encoding the Gal4 DNA-binding domain (called Gal4D), within pcDNA3.1Gal4D-V5, through the BamHI/EcoRI cloning site [43]. Two different types of chimaeras of Nrf1 with Nrf2 were created, one of which were engineered by replacing the *BamHI/EcoRI* fragment (encoding aa 1–328 of Nrf2) with various length cDNA encoding the N-terminal aa 1–607 of Nrf1 (N607) to yield N607:C270^Nrf2^, Nrf1^N607^:C270^Nrf2^ and their mutants (in which C270^Nrf2^ represents the C-terminal 270 aa of Nrf2 being retained); another were made by inserting additional three fragments encoding the C-terminal 112 aa of Nrf1 (C112^Nrf1^) into the *XhoI/XbaI* sites situated immediately downstream of the entire Nrf2-coding pcDNA3.1/V5 Bis B vector to produce Nrf2:C112^Nrf1^ and its mutants. The fidelity of all cDNA products was confirmed by sequencing.

### Cell culture, transfection, and reporter gene assays

Unless otherwise indicated, monkey kidney COS-1 cells (3×10^5^, which had been previously purchased from ATCC and maintained in our laboratory) were seeded in 6-well plates and grown for 24 h in Dulbecco's Modified Eagle Medium (DMEM) contain 25 mM glucose and 10% foetal bovine serum (FBS). After reaching 70% confluence, the cells were transfected with a Lipofectamine 2000 (Invitrogen) mixture that contained an expression construct for wild-type Nrf1 or a mutant protein, together with each of *P_-1061/_Nqo1*-Luc, *P_SV40_Nqo1*-ARE-Luc, *P_SV40_GSTA2*-6×ARE-Luc or *P_TK_UAS*×4-Luc [12], [43], along with pcDNA4 HisMax/*lacZ* encoding β-galactosidase (β-gal) that was used as a control for transfection efficiency. Additional reporter genes lacking the ARE sequence were used as negative controls. Luciferase activity was measured approximately 36 h after transfection with an expression vector for Nrf1, Nrf1-Nrf2, Gal4-Nrf1, or their mutants and was calculated as fold change (mean ± S.D) relative to 1.0 (of the background activity, i.e. obtained following co-transfection of an empty pcDNA3.1/V5 His B vector and an ARE-driven reporter after subtraction of the non-specific value from co-transfecting an empty pcDNA3.1/V5 His B vector and a non-ARE-containing reporter). The data presented each represent at least three independent experiments undertaken on separate occasions that were each performed in triplicate. Differences in their activities were subjected to statistical analyses.

### Real-time qPCR analysis of endogenous genes in cells expressing distinct Nrf1 isoforms or cognate small-interfering RNA (siRNA)

The human embryonic kidney (HEK)-293T cells (that had been previously purchased from ATCC and maintained in our laboratory) were cultured in DMEM supplemented with 5 mmol/L glutamine, 10% (v/v) FBS, 100 units/ ml of either of penicillin and streptomycin) in the 37°C incubator with 5% CO2. The cells had been tranfected with a Lipofectamine 2000 mixture that contained each of expression construct for distinct Nrf1 isoforms, along with an empty pcDNA3 vector (as a blank control) or an Nrf1-target siRNA (5′-CCCAGCAAUUCUACCAGCCUCAACU-3′) to knock down the endogenous expression) alongside with a scramble siRNA (5′-UUCUCCGAACGUGUCACG-3′); both siRNA sequences were synthesized by Genepharma (Shanghai China). At 48 h after transfection, these cells were harvested and then subjected to extraction of total RNAs, which were subsequently reverse-transcripted into a single-stranded cDNAs as temples of real-time qPCR reactions using the Perfect Real-Time and SYBR Premix Ex-Taq kit), according to manufacturers’ protocols (TaKaRa, Dalian, China). The expression levels of Nrf1 and its target genes in the cells that had been transfected with the vehicle plasmid or the scramble siRNA were given the value of 1.0, and other data were calculated as a ratio of this value and shown as a fold change (mean ± S.D). Notably, mouse *Nrf1^−/−^* embryonic fibroblasts (a gift from Dr. Akira Kobayashi with Doshisha University, Japan) had been grown in DMEM containing 10% (v/v) foetal calf serum, 10 µg/ml of insulin, 5.6 µg/ml transferrin, 6.7 ng/ml of sodium selenite, 0.25% (w/v) NaHCO3 and 100 units/ ml of penicillin/streptomycin), and then were allowed to restore distinct Nrf1 isoforms by transfecting their expression constructs alone or in various combinations, followed by real-time qPCR analysis of Nrf1-target gene expression. The data presented each represent at least three independent experiments, each of which were performed in triplicate, followed by statistical analysis of significant differences.

### Live-cell imaging combined with in vivo membrane protease protection assays

COS-1 cells expressing each of green fluorescent protein (GFP) fusion proteins with the CTD of Nrf1 or its mutants and ER/DsRed were subjected to *in vivo* membrane protease protection assays, along with live-cell imaging as reported previously [52]. Briefly, COS-1 cells were permeabilized by digitonin (20 µg/ml) for 10 min, and were then allowed the *in vivo* membrane protection reactions against digestion by PK (50 µg/ml) for various lengths of time before addition of 0.1% (v/v) Triton X-100 (TX). In the experimental course, the live-cell images were acquired every one min under a 40× objective lens mounted on the Leica DMI 6000 green and red fluorescence microscopes equipped with a high-sensitivity Hamamatsh ORCA-ER camera, cell environment control units (at 37°C in 5% CO_2_ culture conditions) and a definitive focus module. Relative fluorescence units were measured with Simulator SP5 Multi-Detection system for GFP with 488-nm excitation and 507-nm emission and for DsRed with 570-nm excitation and 50-nm emission. The intensity of the fluorescent signals lined is quantified automatically according to the instrumental software.

### Immunocytochemistry followed by confocal imaging

COS-1 cells had been transfected with with expression constructs for V5-tagged Nrf1, GFP-CTD or their mutants (1.3 µg DNA of each) and allowed to recover for 24 h as described elsewhere [43]. Thereafter, subcellular location of proteins was examined by immunocytochemistry followed by confocal imaging. Fluorescein isothiocyanate (FITC)-labelled second antibody was used to locate V5-tagged proteins. Nuclear DNA was stained by 4′,6-Diamidino-2-Phenylindole (DAPI). The ER/DsRed (an ER-localized red fluorescent protein marker) gave a red image in the ER. The merge signal represents the results obtained when the three images were superimposed. The corresponding quantitative data shown here were calculated by determining the percentage of cells (at least 200 cells counted) in which the extra-nuclear stain, i.e. cytoplasmic plus ER (called simply C) was greater than or equal to the nuclear stain (called N), as opposed to the percentage of cells in which the extra-nuclear stain was less than the nuclear stain. Bar = 20 µm.

### Western blotting

Equal amounts of protein prepared from cell lysates loaded into each electrophoretic well were monitored by β-gal activity, and visualized by immunoblotting with distinct antibodies against V5, Xpress or Nrf2. Glyceraldehyde-3-phosphate dehydrogenase (GADPH) or β-Actin served as an internal control to verify amounts of proteins that were loaded in each well. On some occasions, nitrocellulose membranes that had already been blotted with an antibody were washed for 30 min with stripping buffer before being re-probed with an additional primary antibody [53].

### Bioinformatic analysis

The membrane-topology of Nrf1 was predicted using several bioinformatic algorithms, including the TopPred (http://mobyle. pasteur.fr/cgi-bin/portal.py?form = toppred), HeliQuest (http://heliquest.ipmc.cnrs.fr/) and AmphipaSeek (http://npsa-pbil.ibcp.fr/cgi-bin/npsaautomat.pl?page=/NPSA/npsaamphipaseek.html) programmes. The T-Coffee program was employed to align Nrf1 amino acid sequences with those of its orthologues.

### Statistical analysis

The statistical significance of changes in Nrf1 activity and its target gene expression was determined using the Student’s *t* test or *M*ultiple *An*alysis *o*f *Va*riations (MANOVA). The data presented herein are shown as a fold change (mean ± S.D), each of which represents at least 3 independent experiments undertaken on separate occasions that were each performed in triplicate.

## Results

### The transactivation activity of Nrf1 is negatively regulated by its CTD

**Figure 2:**
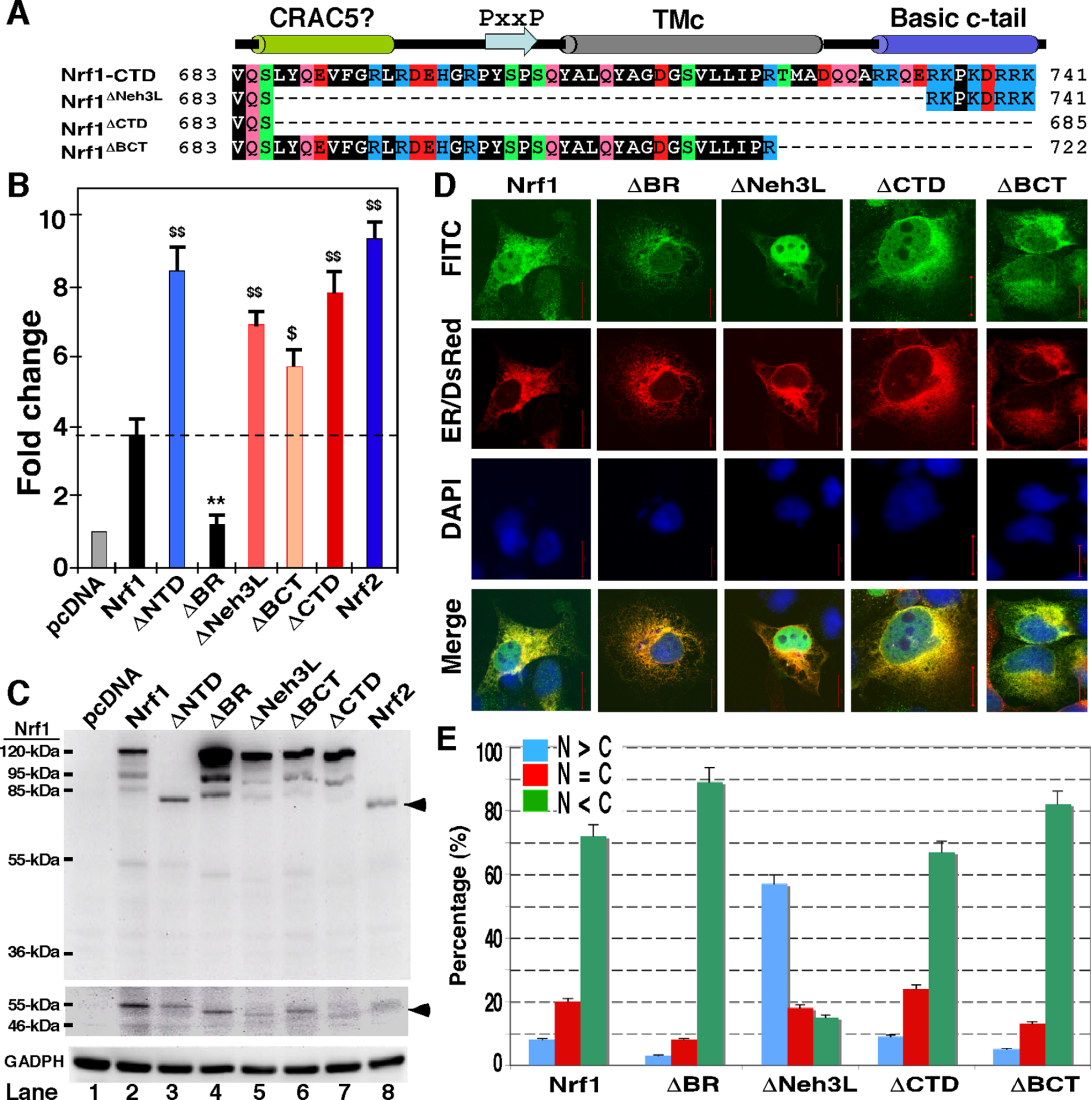
Nrf1 is negatively regulated by its CTD. (**A**) Diagrammatic representation of various lengths of CTD in Nrf1 and its mutants. The putative secondary structure of discrete regions within CTD is shown (*upper cartoon*). (**B**) Luciferase activity was measured from COS-1 cells had been transfected with each of expression constructs for Nrf1 or its mutants (1.2 µg), together with *P_SV40_Nqo1*-ARE-Luc (0.6 µg) and β-gal plasmid (0.2 µg), and allowed to recover in fresh media for an additional 24 h before lysis. The data were calculated as a fold change (mean ± S.D) of transactivation by Nrf1 or its mutants. Significant increases ($, p<0.05 and $$, p<0.001, n = 9) and decreases (**, p<0.001, n = 9) in activity relatively to wild-type Nrf1 are indicated. (**C**) The above-prepared cell lysates (30 µg of protein) were resolved by gradient LDS/NuPAGE containing 4–12% polyacrylamide in a Bis-Tris buffer system and visualized by western blotting with antibody against the V5 epitope. The amount of proteins loaded into each electrophoresis sample well was adjusted to ensure equal loading of β-gal activity. An arrow indicates Nrf2 with a molecular mass of ∼80-kDa estimated (*upper panel*), whereas another arrow points to the brightly-contrasted band of ∼55-kDa Nrf1β (*middle panel*) that was cropped from the same gel as shown in the upper panel. GAPDH served as a protein-loading control (*lower panel*). It is notable that the same protein exhibits distinct mobility on different electrophoretic gels in different running buffer systems (*cf.* Figs. 2***C*** with 5***C***). (**D**) COS-1 cells were co-transfected with 1.3 µg DNA of each of the above-described expression constructs and 0.2 µg of the ER/DsRed plasmid, and then allowed to recover from transfection for 24 h before being fixed. Subcellular location of proteins was examined by immunocytochemistry followed by confocal imaging. FITC-labelled second antibody was used to locate V5-tagged proteins. Nuclear DNA was stained by DAPI. The ER/DsRed gave a red image in the ER. The merge signal represents the results obtained when the three images were superimposed. (**E**) The quantitative data of imaging (corresponding to those shown in panel **D**) were calculated by determining the percentage of cells (at least 200 cells counted) in which the extra-nuclear stain, i.e. cytoplasmic plus ER (called simply C) was greater than or equal to the nuclear stain (called N), as opposed to the percentage of cells in which the extra-nuclear stain was less than the nuclear stain. Bar = 20 µm.

The CTD of Nrf1 is highly conserved amongst different vertebrate species (Fig. S1), and shares 50% sequence identity and 70% similarity with the Neh3 domain of Nrf2, as well as equivalents of other CNC-bZIP factors (Fig. 1B). Although the Neh3 domain was suggested to be required for Nrf2 activity [37], our ARE-driven reporter assay of COS-1 cells (that had been transfected with an expression construct for wild-type Nrf1 or its mutants, illustrated in Fig. 2A) showed evidence to the contrary, that the Neh3L region within CTD contributes to negative regulation of Nrf1 activity (Fig. 2B). Indeed, Nrf1-mediated gene transactivation was significantly increased by deletion of the entire CTD (to yield Nrf1^ΔCTD^) or its portions (to yield Nrf1^ΔNeh3L^ and Nrf1^ΔBCT^, lacking aa 686–733 or 723–741, respectively), to an extent close to that obtained from an NTD-deleted mutant (i.e. Nrf1^ΔNTD^ lacking aa 1–124 within its NTD), and even near to that of the wild-type Nrf2. Western blotting revealed that Nrf1^ΔNTD^ has an electrophoretic mobility similar to that of Nrf2 (80-kDa on NuPAGE gels) (Fig. 2C, *upper panel, lanes 3 vs 8*). When compared with wild-type Nrf1, slight faster migrations of mutant glycoproteins (∼120-kDa), deglycoproteins (∼95-kDa) and cleaved polypeptides (∼85-kDa) were observed in Nrf1^ΔCTD^, Nrf1^ΔBCT^, Nrf1^ΔNeh3L^ and Nrf1^ΔCTD^ (Fig. 2C, *upper panel, cf. lanes 5–7 with 2*). In addition, these three mutants also appeared to reduce generation of the cleaved 85-kDa Nrf1 polypeptide (Fig. 2C, *upper panel*) and/or the shorter Nrf1β/LCR-F1 of 55-kDa (Fig. 2C, *middle panel*), as compared to their wild-type equivalents (*cf. lanes 5–7 with 2*).

Intriguingly, no marked changes in the subcellular location of Nrf1^ΔCTD^ and Nrf1^ΔBCT^ were visualised by confocal microscopy, when compared with the wild-type Nrf1 showing primarily extra-nuclear staining that superimposed with the red fluorescent signal from the ER/DsRed maker (Fig. 2, D and E), although previous subcellular fractionation data of the 120-kDa Nrf1^ΔCTD^ (i.e. Nrf1^Δ686–741^) mutant protein revealed a modest enhancement of its recovery in ER fractions relative to wild-type glycoprotein [12] (indicating that integration of Nrf1 within ER is unaffected by loss of its CTD). By contrast, the Nrf1^ΔNeh3L^ mutant (in which a classic bipartite nuclear localization signal (NLS) sequence ^625^RDIRRRGKNKMAAQNCRKRKL^645^ is retained) enabled a significant increase in the nuclear staining of ∼55% cells observed (Fig. 2, D and E). By contrast, deletion of the basic region encompassing the NLS (to yield the Nrf1^ΔBR^ mutant) caused a significant decrease in the nuclear staining of ∼3% cells, as revealed by a relative enhancement in either the cytoplasmic staining of ∼90% cells (Fig. 2, D and E) or the abundance of Nrf1^ΔBR^ mutant proteins between 120-kDa and 85-kDa (Fig. 2C, *upper panel*). Such a decrease in the nuclear localization of Nrf1^ΔBR^ resulted in a reduction in the reporter gene activity to approximately 40% of that obtained from the wild-type Nrf1 (Fig. 2B). Therefore, the transactivation activity of Nrf1 (and possibly its protein stability) is monitored by its NLS-containing basic region, enabling the CNC-bZIP protein to translocate the nucleus before binding ARE sequences in target genes. By contrast, the basic c-tail ^730^RRQERKPKDRRK^741^ within CTD is not required for the nuclear localization of Nrf1 rather than for its primary cytoplasmic location.

### Attachment of the CTD of Nrf1 to GFP enables the chimaeras to associate with the ER

**Figure 3:**
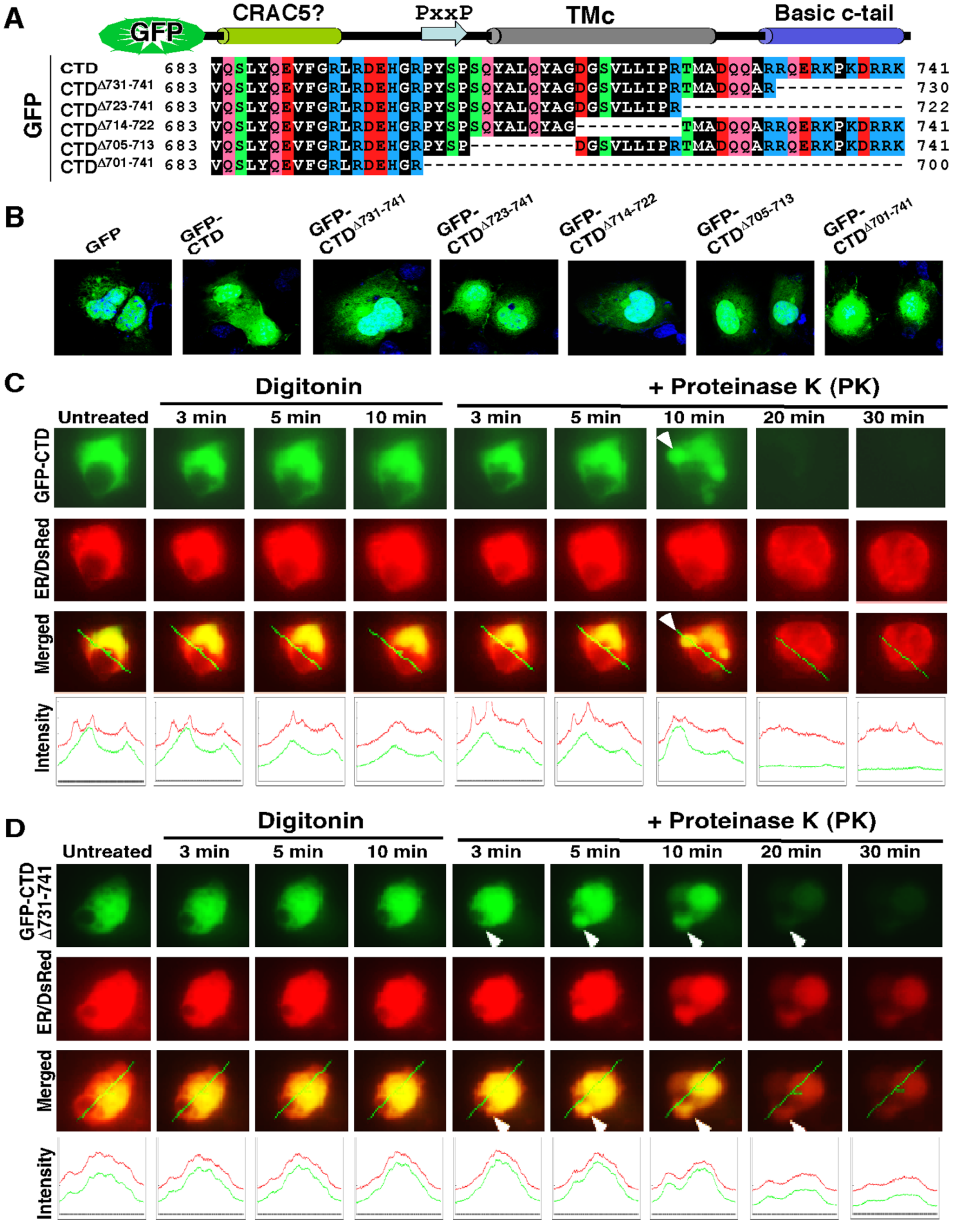
Imaging of fixed and live cells expressing GFP fusion protein with CTD of Nrf1 or its mutants. (**A**) Schematic of Six expression constructs for the GFP-CTD fusion protein and its mutants; these fusion proteins have been created by attachment of various lengths of CTD of Nrf1 to the C-terminus of GFP. (**B**) These indicated expression constructs each were transfected into COS-1 cells for 6 h. The cells were then allowed to recover from transfection in fresh medium for 18 h before being fixed by 4% paraformaldehyde and stained for the nuclear DNA by DAPI. The green signals from GFP were observed under confocal microscope and merged with the DNA-staining images. (**C** and **D**) Live-cell imaging of GFP-CTD and its mutant GFP-CTD^Δ731–741^(lacking its basic c-tail). COS-1 cells had been transfected with expression constructs for either GFP-CTD (***C***) or GFP-CTD^Δ731–741^ (***D***), together with the ER/DsRed marker, before being subjected to real-time live-cell imaging combined with the *in vivo* membrane protease protection assay. The cells were permeabilized by digitonin 20 µg/ml) for 10 min, before being co-incubated with PK (50 µg/ml) for 30 min. In the time course, real-time images were acquired using the Leica DMI-6000 microscopy system. The merged images of GFP with ER/DsRed are placed (on *the third raw of panels*), whereas changes in the intensity of their signals are shown graphically (*bottom*). Overall, the images shown herein are a representative of at least three independent experiments undertaken on separate occasions that were each performed in triplicate (n = 9). The *arrow* indicates a ‘hernia-like’ vesicle protruded from the cytoplasm.

To address whether the basic c-tail ^730^RRQERKPKDRRK^741^ adjacent to the amphipathic Neh3L region of Nrf1 is required for its association with the ER or its localization in the nucleus, we attached the entire CTD and its portions to the C-terminus of GFP (giving GFP-CTD and mutant constructs, Fig. 3A) and then cotransfected them with an ER-resident marker into COS-1 cells, before being subjected to fixed-cell microscopy experiments. When compared with the primary nuclear imaging of GFP alone (as theoretically a protein with molecule mass of less than 40 kDa can easily translocate the nucleus), an increase in the extra-nuclear subcellular distribution of the fusion protein GFP-CTD appeared to be similar to that of GFP-CTD^Δ701–741^ (Fig. 2B). By contrast, CTD^Δ731–741^, CTD^Δ723–741^, CTD^Δ714–722^ or CTD^Δ705–713^ gave a slight increase in the nuclear green staining of GFP fusion protein, relative to the extra-nuclear staining (Fig. 3B). This observation indicates that the basic c-tail of Nrf1 does not act as functional NLS, and give rise to a possibility that the basic c-tail adjoining the amphipathic TMc region enables the GFP-CTD fusion protein to be retained in putative extra-nuclear subcellular compartments.

**Figure 4:**
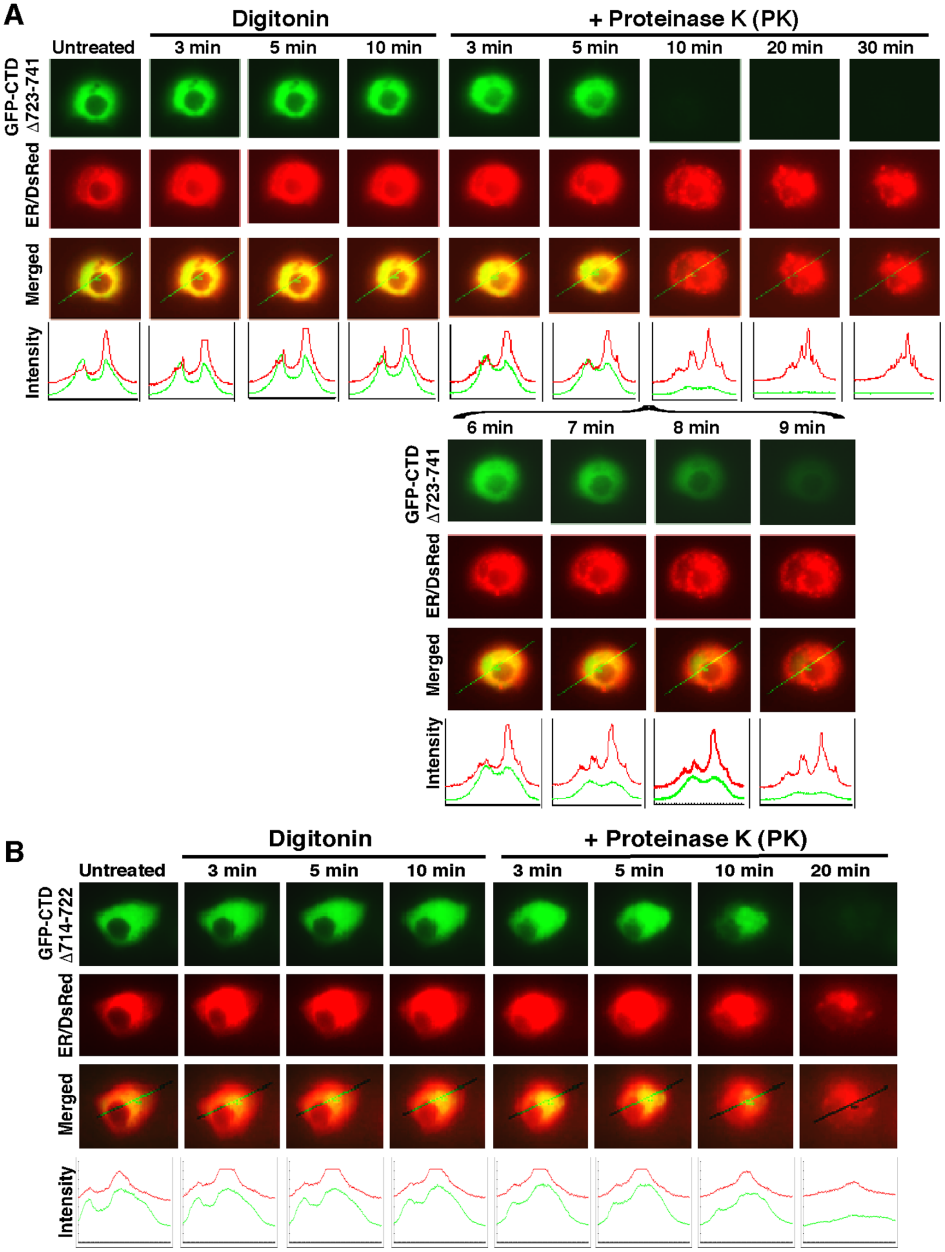
Live-cell imaging of both mutants GFP-CTD^Δ723 −741^ and GFP-CTD^Δ714–722^. COS-1 cells co-expressing either GFP-CTD^Δ723 −741^ (**A**) or GFP-CTD^Δ714–722^ (**B**), along with the ER/DsRed marker, were subjected to live-cell imaging combined with the *in vivo* membrane protease protection assay, as described above in Figure 3. The images shown herein are a representative of at least three independent experiments undertaken on separate occasions that were each performed in triplicate (n = 9).

To test this hypothesis, we performed live-cell imaging of GFP-CTD and its mutants combined with *in* *vivo* membrane protease protection assays, in order to determine whether the extra-nuclear-localized proteins is capable of being either associated with the cytoplasmic side of the ER or dislocated from the lumen to the cytoplasmic side of the membrane. In these experiments, COS-1 cells that had been transfected with expression constructs for CTD-GFP or its mutants together with ER/DsRed were first pre-treated for 10 min with digitonin to pemeabilize cellular membranes before being challenged with PK for 3–90 min (in the presence of digitonin) to digest cytoplasmic proteins. Thus we envisaged that if extra-nuclear GFP fusion protein was not tethered to membranes, it would diffuse rapidly into the extracellular compartments while the plasma membrane was disrupted by digitonin; otherwise, if the GFP fusion protein was associated with the ER membranes or transferred from the lumen to the cytoplasmic side of the membrane, it would become vulnerable to digestion by PK. As anticipated, Figure 3 (C and D) showed the green fluorescent signal from extra-nuclear CTD-GFP and GFP:CTD^Δ731–741^ appeared to be partially superimposed upon red fluorescent images presented by ER/DsRed. No apparent change in the intensity of their green signals was observed within 10 min following treatment of the cells with digitonin (Fig. 3, C and D), but subsequently exposure to PK allowed the GFP fusion protein to be digested gradually from 3 min to 20 min before being destroyed completely. Similarly, live-cell imaging of GFP-CTD^Δ723–741^and GFP-CTD^Δ714–722^ revealed that the extra-nuclear subcellular proteins appeared to be unaffected by digitonin, and most of them were completely digested by PK within 10 min (Fig. 4A) or ∼20 min (Fig. 4B). By contrast, GFP-CTD^Δ701–741^ (retaining a cholesterol recognition/amino acid consensus (CRAC5) motif only) gave strong staining in both the nuclear and extra-nuclear compartments (Fig. S2A), in which it appeared insensitive to PK until the protease incubation time was extended to 45 min, and then it was digested to disappear by 48 min (Fig. S2B). Taken together, these results indicate that an extra-nuclear subcellular fraction of GFP-CTD could be tethered to the cytoplasmic side of ER membranes or be dynamically dislocated into cytoplasmic subcellular compartments.

### LCR-F1/Nrf1β is a weak activator, whilst Nrf1γ (or Nrf1δ) acts as a dominant-negative inhibitor

**Figure 5:**
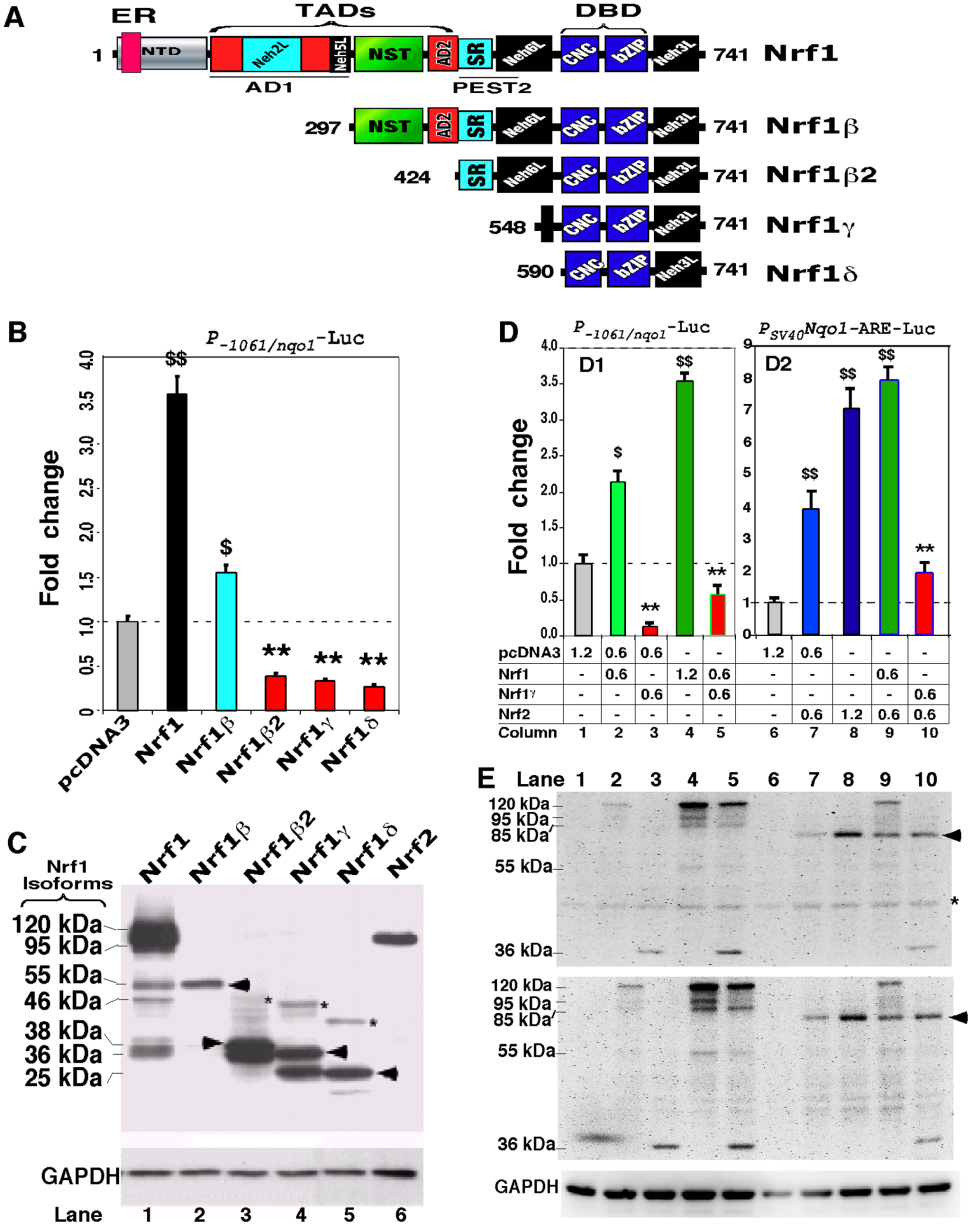
Opposing regulation of ARE-driven reporter genes by distinct Nrf1 isoforms. (**A**) Schematic shows structural domains of five different isoforms of Nrf1. Locations of ER-targeting signal, AD1 and PEST2 are also indicated within distinct domains. (**B**) Shows luciferase reporter gene activity measured from COS-1 cells that had been co-transfected with 1.2 µg of each expression construct for Nrf1 isoforms, together with 0.6 µg of *P_-1061/_nqo1*-Luc (that is driven by the 1061-bp promoter of *Nqo1*) and 0.2 µg of β-gal plasmid. The data were calculated as a fold change (mean ± S.D) of transactivation by distinct Nrf1 isoforms. Significant increases ($, p<0.05 and $$, p<0.001, n = 9) and decreases (**, p<0.001, n = 9) in activity were calculated relatively to the background activity (obtained from transfection of cells with an empty pcDNA3 with reporter plasmids). (**C**) Total lysates of COS-1 cells expressing each of Nrf1 isoforms or Nrf2 were resolved by 12% SDS-PAGE in a Bis-Tris buffer system and visualized by immunoblotting with the V5 antibody. The position of migration of the V5-tagged polypeptide was estimated to be 120, 95, 55, 46, 38, 36 and 25 kDa, and GAPDH was used as an internal control to verify amounts of proteins loaded into each electrophoretic well. (**D**) Nrf1γ inhibits transactivation of ARE-driven genes by Nrf1 or Nrf2. COS-1 cells were co-transfected with indicated amounts of expression constructs for Nrf1, Nrf1γ and/or Nrf2, together with 0.6 µg of *P_-1061/_nqo1*-Luc (***D1***) or *P_SV40_Nqo1*-ARE-Luc (***D2***) and 0.2 µg of β-gal plasmid. Thereafter, luciferase activity was measured and is shown as a fold change (mean ± S.D). Significant increases ($, p<0.05 and $$, p<0.001, n = 9) and decreases (**, p<0.001, n = 9) in activity relatively to the background activity are indicated. (**E**) Total lysates of COS-1 cells co-transfected with expression constructs for Nrf1, Nrf1γ and/or Nrf2 alone or in combination (as indicated corresponding to those in panel D) was subject to separation by 4–12% LDS/NuPAGE in a Bis-Tris buffer system. The *upper two panels* represent similar images from different independent gels, on which location of Nrf2 migration is *arrowed*, whilst a non-specific protein band is *starred* (*). The position of the V5-tagged Nrf1 polypeptides of 120, 95, 85, 55, and 36 kDa is indicated. It is notable that the same proteins exhibit distinct mobilities on different electrophoric gels in different running buffer systems (*cf.* ***C*** *with* ***E***).

To identify the biological significance of the CTD within distinct Nrf1 isoforms, we created a series of expression constructs for five distinct isoforms (Fig. 5A) into COS-1 cells, in order to determine which isoforms are activators or dominant-negative isoforms. The *P_SV40_Nqo1-ARE-Luc* reporter gene assay showed that the shorter LCR-F1/Nrf1β exhibited a ∼1.6-fold transactivation activity against the background value of 1 (which was measured from transfection of cells with an empty pcDNA3 vector; it is notable that the baseline reporter activity is theoretically deduced to be mediated by endogenous Nrf1, Nrf2 and/or other transcription factors that enable to bind the ARE-driven gene) (Fig. 5B), as it was expressed as a major single band of 55 kDa (Fig. 5C, *lane 2*). By contrast, over-expression of Nrf1β_2_ (aa 424–741), Nrf1γ (aa 548–741) and Nrf1δ (aa 590–741) significantly diminished ARE-driven reporter gene activity from 40% to 20% of the background level (Fig. 5B). All the three small isoforms of Nrf1 were expressed as major proteins with molecular masses of between 38-kDa and 25-kDa, as migrated on the general electrophoretic gels containing 12% polyacrymide in an SDS Bis-Tris buffer system (pH 8.9), which were smeared with high mass polypeptide ladders from 46-kDa to 38-kDa (Fig, 5C). Together, these results indicate that the 55-kDa Nrf1β is *per se* a weaker activator than the full-length Nrf1, whereas smaller isoforms Nrf1β_2_, Nrf1γ and Nrf1δ exert differentially dominant-negative effects on its activity.

**Figure 6:**
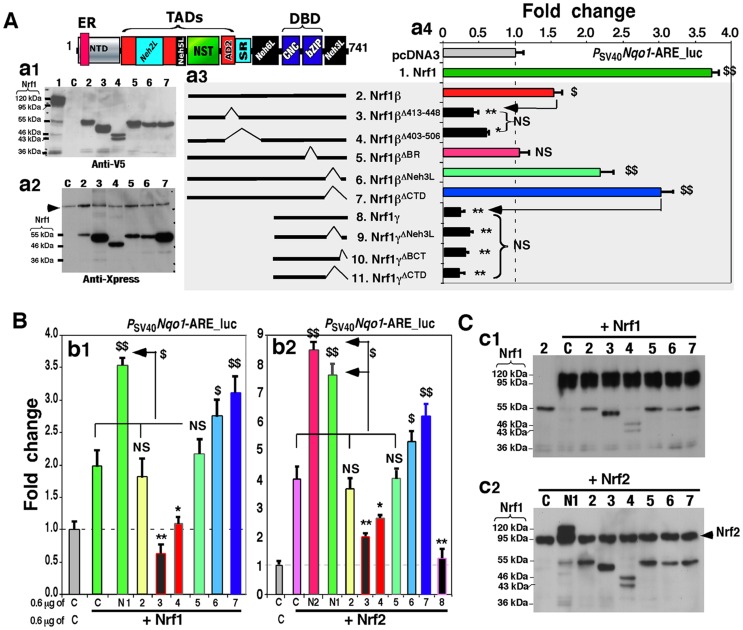
The weak activator Nrf1β/LCR-F1 is negatively regulated by its CTD. (**A**) The *middle* schematic representation of Nrf1β, Nrf1γ and their deletion mutants lacking various lengths of aa 297–741 of Nrf1 (***a3***). The contributions of the deleted regions to changes in the activity of Nrf1β and Nrf1γ, when compared with the background value, were examined using the *P_SV40_Nqo1*-ARE-Luc reporter assay as described above. The *right panel* shows ARE-driven luciferase activity (***a4***) that was measured from COS-1 cells that had been co-transfected with each of numbered expression constructs and reporter plasmids. The data are shown as a fold change (mean ± S.D), and significant increases ($, p<0.05 and $$, p<0.001, n = 9) and decreases (*p<0.05, **p<0.001, n = 9) are indicated, relatively to the background value from transfection with an empty pcDNA3 control vector alone (*C*). The *left two panels* show western blotting of some of the above-transfected cell lysates with antibodies against either V5 (***a1***) or Xpress (***a2***). In addition, a non-specific protein-band is indicated (by *arrow*). The amount of protein applied to each polyacrylamide gel sample well was adjusted to ensure equal loading of β-gal activity. (**B**) COS-1 cells were co-transfected with each of the above-numbered expression constructs for Nrf1β, Nrf1γ and their mutants, together with an expression vector for wild-type Nrf1 (**N1**) or Nrf2 (**N2**), *P_SV40_Nqo1*-ARE-Luc and β-gal plasmids. The cells were allowed to recover from transfection for 24 h before luciferase activity was measured**.** The data are shown as a fold change (mean ± S.D) of ARE-driven gene activity when compared with the background (value of 1.0). Significant increases ($, p<0.05 and $$, p<0.001, n = 9) and decreases (*p<0.05, **p<0.001, n = 9) are indicated. (**C**) The above-prepared cell lysates (***b1*** and ***b2***) were resolved using 4–12% LDS/NuPAGE and visualized by western blotting with V5 antibody (***c1*** and ***c2***). The electrophoresis band representing Nrf2 is indicated ((by *arrow*). The amount of protein loaded to each electrophoretic well was adjusted to ensure equal loading of β-gal activity.

Further examination of co-transfected cells by *P_-1061_nqo1-Luc* and *P_SV40_Nqo1-ARE-Luc* reporter gene assays revealed that forced expression of Nrf1γ caused a significant decrease in the transactivation activity of the full-length Nrf1 to ∼60% of the background level (Fig. 5D1, *column 5*), whilst the activity of Nrf1 appeared to be unaffected by expression of LCR-F1/Nrf1β (as shown in Fig. 6B1, *left panel*). In the parallel experiments, Nrf1γ also dominantly repressed Nrf2-mediated reporter gene activity to ∼25% of its original transactivation activity obtained from cells that had been transfected with an Nrf2 expression construct alone (Fig. 5D2, *column 10*). In addition, western blotting of these proteins that had been separated by 4–12% NuPAGE in an LDS Bis-Tris buffer system (pH 7.0) revealed no changes the abundance of Nrf1 and Nrf2 when co-transfected with Nrf1γ (Fig. 5E, *upper two panels*). By contrast, the transactivation activity of Nrf2 was neither suppressed by LCR-F1/Nrf1β (Fig. 6B2, *right panel*) nor influenced by the full-length Nrf1 (Fig. 5D2, *column 9*). Moreover, expression of Nrf1 and Nrf2 is also unaffected by LCR-F1/Nrf1β (Fig. 6C). Collectively, these findings demonstrate that Nrf1γ, but not LCR-F1/Nrf1β, is a *bona fide* dominant-negative form competitively against the intact wild-type factors Nrf1 and Nrf2.

### LCR-F1/Nrf1β is positively and negatively regulated by its AD2 and CTD, respectively

**Figure 7:**
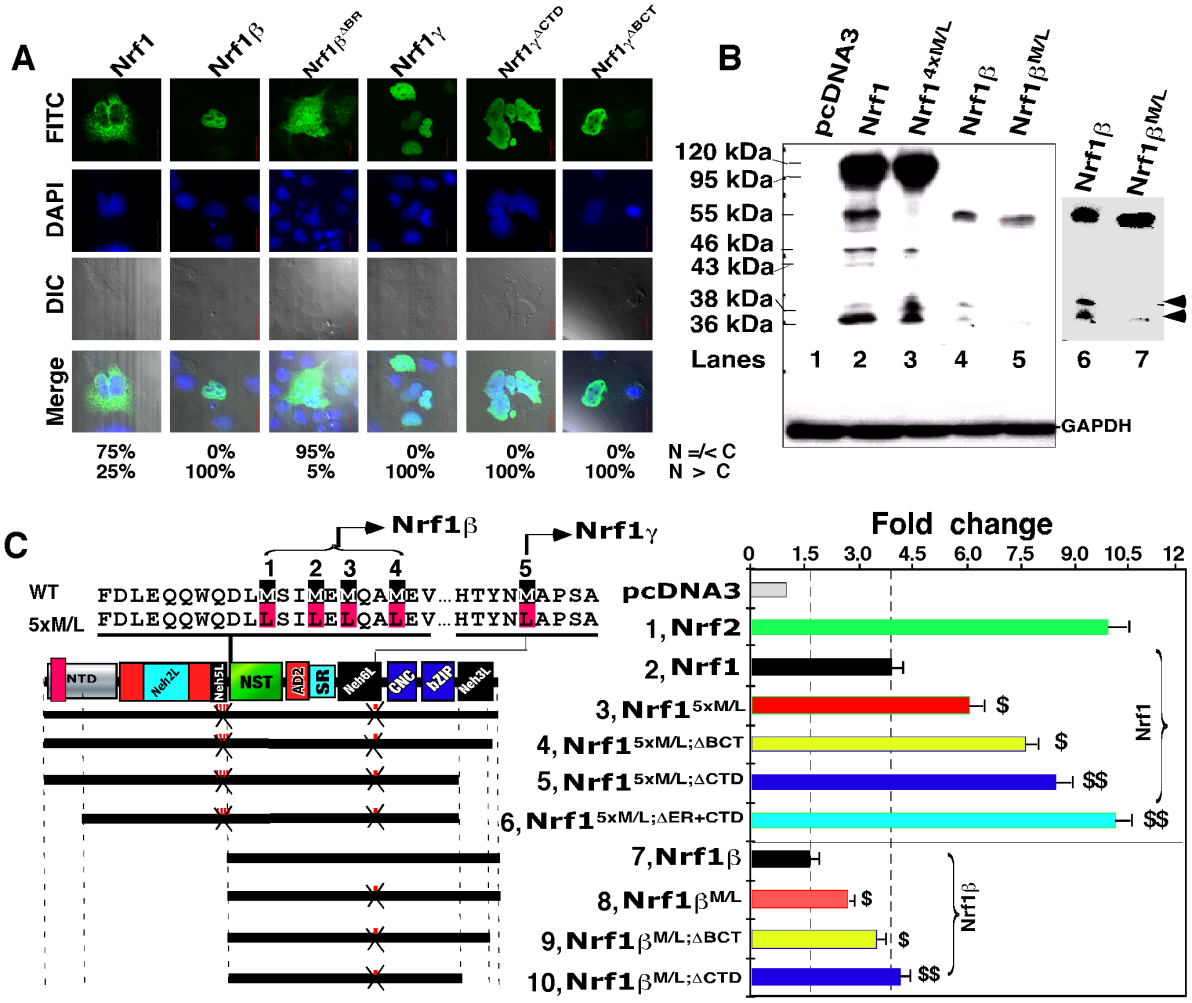
Blockage of Nrf1γ results an increase in the transactivation activity of Nrf1 and Nrf1β/LCR-F1. (**A**) Confocal imaging of COS-1 cells that had been transfected with 1.3 µg DNA of each expression construct for Nrf1, Nrf1β and Nrf1γ or mutants, before their subcellular locations were then examined by immunocytochemistry with FITC-labelled second antibody in order to locate V5-tagged proteins. Nuclear DNA was stained by DAPI. The merge signal represents the results obtained when the two images were superimposed with DIC from normal light microscopy. Bar = 20 µm. The quantitative data (*bottom*) were calculated as described in Figure 2D. (**B**) Western blotting of COS-1 cells that had been transfected with the indicated expression constructs for V5-tagged Nrf1, Nrf1β and Nrf1γ and their point mutants (Met into Leu, *below*). The *right panel* shows that the same gel as *the left panel* was exposed to X-ray for a little longer time. Two bands representing the 36-kDa Nrf1γ and a 38-kDa polypeptide are indicated (*arrows*). GAPDH served as an internal control to verify the amount of proteins applied to each electrophoresis well. (**C**) Schematic representation of Nrf1, Nrf1β, and their Met-to-Leu mutants with various deletions. The *upper left panel* shows amino acids adjoining five numbered Met residues; their mRNA codons can be recognized by ribosome for the internal initiation to translate Nrf1β or Nrf1γ. The first four or all five Met-to-Leu mutants were made respectively to yield Nrf1^4xM/L^ and Nrf1^5xM/L^, whilst Nrf1β^ M/L^ contains the fifth Met-to-Leu mutant. Additional deletion mutants were created on the base of Nrf1^5xM/L^ and Nrf1β^M/L^. The *right panel* shows luciferase reporter activity of COS-1 cells that had been transfected with 1.2 µg of each of indicated expression constructs, together with 0.6 µg of *P_SV40_nqo1-ARE*-Luc and 0.2 µg of β-gal plasmids. The data are shown graphically as fold changes (mean ± S.D.) of transactivation by indicated factors. Significant increases ($, p<0.05 and $$, p<0.001, n = 9) in the activity are compared to the activity of the intact Nrf1 or Nrf1β.

Since CTD has the negative effect on the intact full-length Nrf1, we examine whether this domain auto-inhibits LCR-F1/Nrf1β. As expected, an approximately 1.5∼2.0-fold increase in the transactivation activity of LCR-F1/Nrf1β resulted from removal of its essential Neh3L region (in Nrf1β^ΔNeh3L^) or the entire CTD (in Nrf1β^ΔCTD^) (Fig. 6A, *cf. columns 6 *&* 7 with 2*). These two CTD-deficient mutants of LCR-F1/Nrf1β also caused an enhancement in the reporter gene transactivation by Nrf1 or Nrf2 (Fig. 6B, *columns 6 *&* 7*), when compared to the indicated controls. However, removal of either CTD or its portions from Nrf1γ did neither disinhibit its dominant negative effect on ARE-driven gene activity (Fig. 6A, *cf. columns 9–10 with 8*), and nor influence the nuclear location of the smaller isoform (Fig. 7A). These data indicate that CTD is not ascribed to the dominant negation by Nrf1γ, albeit it enables negative regulation of both Nrf1 and LCR-F1/Nrf1β. Furthermore, the activity of LCR-F1/Nrf1β was abolished by loss of the DNA-binding basic region in the Nrf1β^ΔBR^ mutant, but it did not act as a potential dominant-negative competitor against either endogenous or exogenous activities of Nrf1 and Nrf2 (Fig. 6, A and B, *columns 5 vs 2*). This is likely to be associated with a decrease in its nuclear staining to ∼5% of cells examined (Fig. 7A).

To gain an insight into the biological significance of LCR-F1/Nrf1β, we next examined which regions of this isoform contribute to its transactivation activity to positively regulate ARE-driven genes. As shown in Figure 6A (*cf. columns 3 with 2*), the Nrf1β^Δ413–448^ mutant (lacking a major acidic-hydrophobic amphipathic region within AD2) significantly reduced Nrf1β-mediated transactivation activity to ∼40% of the background level. Similarly, the Nrf1β^Δ403–506^ mutant (lacking the entire AD2 together with both SDS1 and SR/PEST2 degrons) exhibited ∼60% of the background activity, though its dominant-negative effect was modestly alleviated as compared with that of Nrf1^Δ413^–^448^ (Fig. 6A, *columns 4 vs 3*). Moreover, both Nrf1β^Δ413–448^ and Nrf1β^Δ403–506^ enabled dominant-negative competition against the wild-type Nrf1 and/or Nrf2 activity to be blunted markedly (Fig. 6B, *columns 3 *&* 4*). In turn, these results demonstrate that AD2, but not the SR domain, contributes to the positive regulation of Nrf1β/LCR-F1. Upon Nrf1β/LCR-F1 co-expression, the abundance of Nrf1 and Nrf2 is unaffected (Fig. 6C).

### Blockage of Nrf1γ expression results in an increase in the activity of Nrf1 and LCR-F1/Nrf1β

Distinct Nrf1 isoforms are proposed to arise from the in-frame translation from an internal ATG codon or the selective post-translational processing of a larger Nrf1 isoform [46], [48]. As shown in Figure 7B, generation of the 55-kDa LCR-F1/Nrf1β was completely abolished by the Nrf1^4xM/L^ mutant (in which the internal translation start codons of Nrf1 at Met^289/292/294/297^ were mutated into leucines), when compared to the wild-type Nrf1 expression (*lanes 3 vs 2*). This mutant also caused an increase in its activity to transactivate *P_sv40_nqo1-ARE*-luc reporter gene by approximately 50% more than that of the wild-type Nrf1 activity [46]. Further examinations revealed that expression of additional two polypeptides of 46-kDa and 43-kDa, besides the 55-kDa LCR-F1/Nrf1β was also, to a lesser extent, prevented by the Nrf1^4xM/L^ mutant (Fig. 7B, *lane 3*). Similarly, two polypeptides of 46-kDa and 43-kDa appeared to be expressed in COS-1 cells that had been transfected with the Nrf1β^Δ403–506^ mutant (which was constitutively tagged by the Xpress or V5 epitopes at its N-terminus and C-terminus, respectively); the 46-kDa protein was detected by western blotting with both antibodies against V5 and Xpress, whilst the 43-kDa protein was only immunoblotted by antibodies against V5 rather than Xpress (Fig. 6A, *lane 4* in *panel a1 vs a2*). These results rise to a possibility that proteolytic processing of the 55-kDa LCR-F1/Nrf1β occurs within and around its NST domain to yield 46-kDa and 43-kDa polypeptides.

The abundance of the dominant-negative Nrf1γ isoforms (i.e. 36-kDa or possibly 38-kDa in Fig. 5C, *lane 4*) appeared to be unaffected by expression of Nrf1^4xM/L^ (Fig. 7B, *lanes 3 vs 2*), but it was obviously diminished by the Nrf1β^M/L^ mutant (in which an additional potential translation start codon at Met^548^ within Nrf1β was mutated into leucine) (Fig. 7B, *lanes 6 vs 7*), implying that Nrf1γ also arises from the internal translation. The failure to produce the dominant-negative Nrf1γ resulted in a ∼2.0-fold increase in the reporter gene transactivation when compared to LCR-F1/Nrf1β-mediated activity (Fig. 7C, *columns 8 vs 7*). Interestingly, a further increase in the transactivation activity of LCR-F1/Nrf1β to ∼4.0-fold activation was determined by deletion of the CTD to yield Nrf1β^M/LDCTD^ (Fig. 7C, *columns 10 vs 8*). Similarly, LCR-F1/Nrf1β activity was also modestly enhanced by the Nrf1β^M/LDBCT^ mutant (*columns 9 vs 8*). Furthermore, a combinative mutant Nrf1^5xM/L^ (in which all five potential ATG start codons at Met^289/292/294/297^ and Met^548^ were mutated into leucines) exhibited a higher activity of ∼6.0-fold induction of *P_SV40_Nqo1-ARE-Luc* reporter gene (Fig. 7C, *column 3*). Excitingly, the activity of Nrf1^5xM/L^ was further increased to approximately 8.0- to 10.0-fold transactivation by loss of its N-terminal ER-targeting sequence and its CTD in Nrf1^5xM/L;ΔER+CTD^ (Fig. 7C, *column 6*). Together, these results demonstrate that LCR-F1/Nrf1β is a weak activator that transactivate Nrf1-target gene expression, because its activity, as well as that of wild-type Nrf1, is negatively regulated by CTD and also is predominantly inhibited by smaller molecular weight dominant-negative forms of between 46-kDa and 25-kDa.

### Chimaeric Gal4/Nrf1 and Gal4/Nrf1β factors are positively regulated by AD2 and NST domains, but are also negatively regulated by the Neh6 domain and its adjoining region

**Figure 8:**
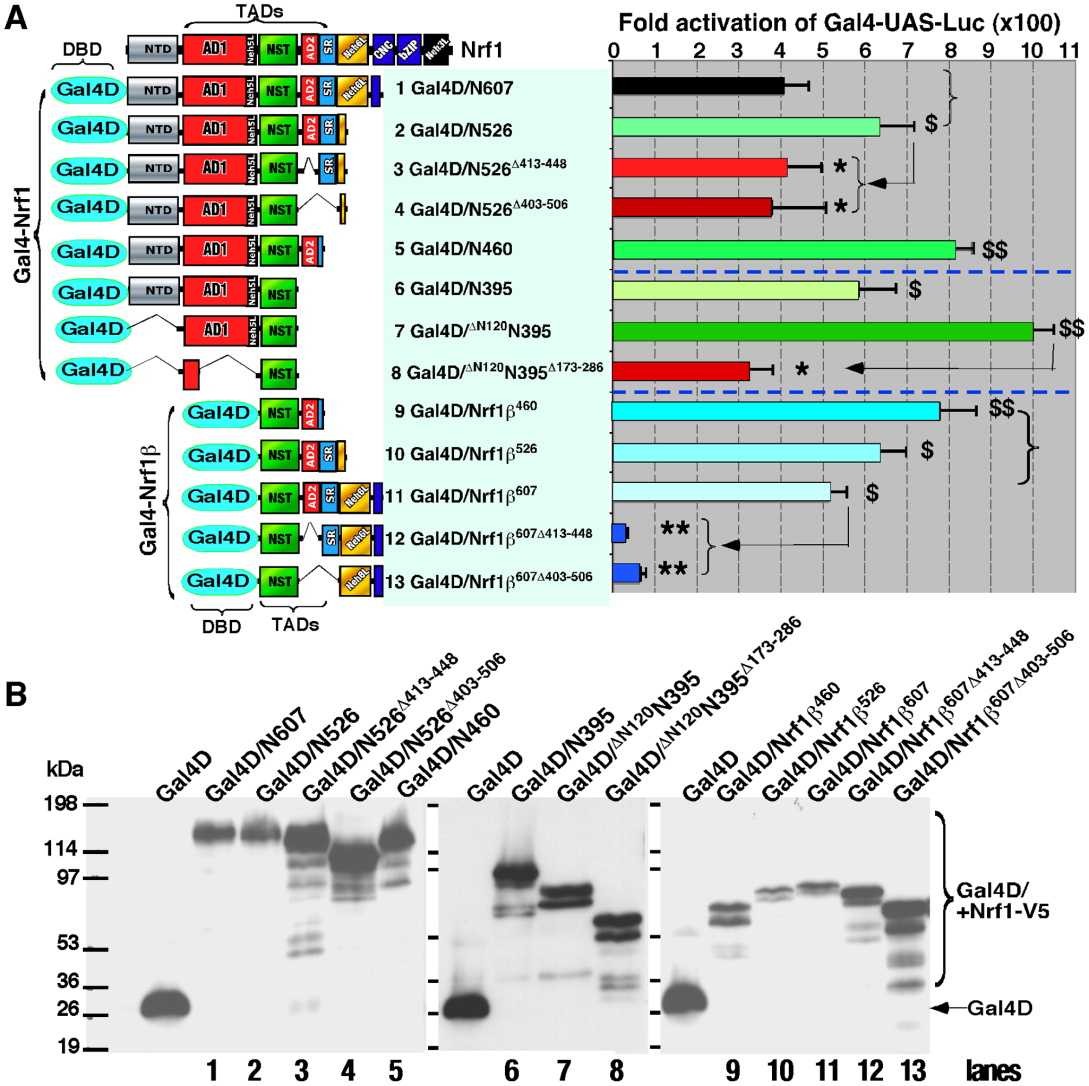
Both AD2 and NST domains positively regulates chimaeric Gal4-Nrf1 and Gal4-Nrf1β factors. (**A**) Schematic representation of expression constructs for Gal4D (Gal4 DNA-binding domain) fusion proteins containing various portions of Nrf1 or Nrf1β (*left panel*). They were created by ligation of their encoding cDNA fragments into the *BamHI/EcoRI* sites of the pcDNA3/Gal4-V5 vector. The *left panel* shows Gal4D-directed reporter activity that was measured from COS-1 cells had been cotransfected with each of indicated expression constructs for the various Gal4D/Nrf1 fusion proteins (1.2 µg), together with *P_TK_UAS*×4*-*Luc (0.6 µg) and b-gal (0.2 µg) plasmids. The data are shown graphically as fold changes (mean ± S.D.) of transactivation by indicated Gal4-fusion factors when compared with the background (value of 1.0). Significant increases ($, p<0.05 and $$, p<0.001, n = 9) and decreases (*p<0.05, **p<0.001, n = 9) in activity relatively to the referenced activity are indicated (*arrows*). (**B**) The above-prepared cell lysates were resolved using 4–12% LDS/NuPAGE and examined by western blotting with V5 antibody. The electrophoretic bands representing free Gal4D and Gal4-Nrf1 fusion proteins are indicated. Samples loaded on each well were calculated to contain equal amounts of β-gal activity.

To determine that AD2 contributes to the transactivation activity of LCR-F1/Nrf1β, we created a series of chimaeric Gal4/Nrf1β factors by fusing Gal4 DNA-binding domain (Gal4D) with the N-termini of various portions of LCR-F1/Nrf1β and its mutants (Fig. 8A, *left panel*). *P_TK_ Gal4-UAS*-Luc reporter assay displayed a ∼510 fold activity mediated by Gal4D/Nrf1β^607^ (containing the NST, AD2, SR/PEST2 and Neh6 regions, *column 11*). Lack of the acidic-hydrophobic amphipathic aa 413–448 of AD2 (in Gal4D/Nrf1β^607Δ413^–^448^) caused a significant decrease in the reporter gene activity to ∼30-fold (Fig. 8A, *column 12*). However, loss of both the entire AD2 and SR/PEST2 regions (in Gal4D/Nrf1β^607Δ403–506^) did not further decrease its transactivation activity (*column 13*). These data indicate that AD2 (and possibly the NST domain) is required for Nrf1β-mediated transactivation, whereas the SR/PEST2 sequence appears to be dispensable for the transactivation activity of Gal4D/Nrf1β^607^. By comparison with Gal4D/Nrf1β^607^, Gal4D/Nrf1β^460^ (retaining NST and AD2) exerted a higher activity to enable ∼790-fold activation of the target reporter gene (Fig. 8A, *column 9*). Gal4D/Nrf1β^526^ (retaining NST, AD2 and SR/PEST2) also showed a ∼630-fold activity, which was more than the ∼510-fold induction by Gal4D/Nrf1β^607^ (Fig. 8A, *columns 10 vs 11*). These observations indicate that Nrf1β^607^-mediated transactivation activity is positively regulated by both its AD2 and NTD regions, but is negatively regulated by its adjacent SR/PEST2 and Neh6L regions. Further comparison of Gal4D/Nrf1β^460^ and Gal4D/^Δ120^N395^Δ173–286^ mutants (only retaining most of the NST domain) revealed that the latter Gal4D/^Δ120^N395^Δ173–286^ still exhibited a ∼320-fold activity to mediate *P_TK_ Gal4-UAS*-Luc reporter gene expression, implying that besides AD2, the NST domain contributes to the positive regulation of Gal4D/Nrf1β^460^ (Fig. 8A, *columns 9 vs 8*).

The molecular basis for the positive regulation of the full-length Nrf1 by its AD2 was herein investigated by measuring differences in between the transcription activities of a series of chimaeric Gal4/Nrf1 factors (in which Gal4D was fused N-terminally with various portions of Nrf1 and its mutants). Gal4D/N460 (in which N460 represents the N-terminal 460 aa of Nrf1, containing AD1, the NST domain, and AD2) exhibited a ∼810-fold transactivation activity, whereas loss of AD2 in Gal4D/N395 (i.e. the N395 portion of Nrf1 contains AD1 and the NST domain) decreased the reporter gene transactivation to ∼590 fold (Fig. 8A, *columns 5 vs 6*). Conversely, a significant increase in the transcriptional activity to a maximal ∼1,000-fold induction was observed by the removal of NTD from Gal4D/N395 (to yield the Gal4D/^Δ120^N395 mutant), and in turn the transactivation activity was markedly decreased to ∼320 fold by deletion of most of AD1 (to yield the Gal4D/^Δ120^N395^Δ173–286^ mutant) (*columns 6 to 8*). These results demonstrate that Gal4/Nrf1 chimaeric factor is positively regulated by its AD1, NST and AD2 regions, and is negatively regulated by its NTD.

Further examinations of Gal4D/N526 (i.e. the N526 portion of Nrf1 contains NTD, AD1, NST, AD2 and SR/PEST2), its mutants Gal4D/N526^Δ413–448^ (lacking the major amphipathic portion of AD2 within Nrf1) and Gal4D/N526^Δ403–506^ (lacking both the entire AD2 and SR/PEST2 regions) (Fig. 8A, *columns 2 to 4*) revealed that AD2, rather than SR/PEST2, is required for transactivation by Gal4/N526. Comparison of Gal4D/N460, Gal4D/N526 and Gal4D/N607 (i.e. the N607 portion of Nrf1 covers its NTD, AD1, NST, AD2, SR/PEST2 and Neh6L regions) showed that ∼810-fold activity of Gal4-driven reporter gene induced by Gal4D/N460 was significantly decreased from ∼620-fold to ∼400-fold changes measured from Gal4D/N526 and Gal4D/N607, respectively (Fig. 8A, *columns 1 to 3*). In addition, our previous publications had reported that Gal4D/N607 exhibited a similar transactivation activity to that of the wild-type full-length Gal4D/Nrf1 [12], [46] (and thus Gal4D/N607 is herein used as a reference basis to create the above-described series of mutants illustrated in Fig. 8A, *left panel*). Collectively, these results demonstrate that both SR/PEST2 and Neh6L regions contribute to negative regulation of chimaeric Gal4D/N607 (or Gal4/Nrf1) factor.

### Both AD2 and NST domains contribute to up-regulation of Nrf2-target gene by its chimaeras N607:C270^Nrf2^ and Nrf1β^N607^:C270^Nrf2^

**Figure 9:**
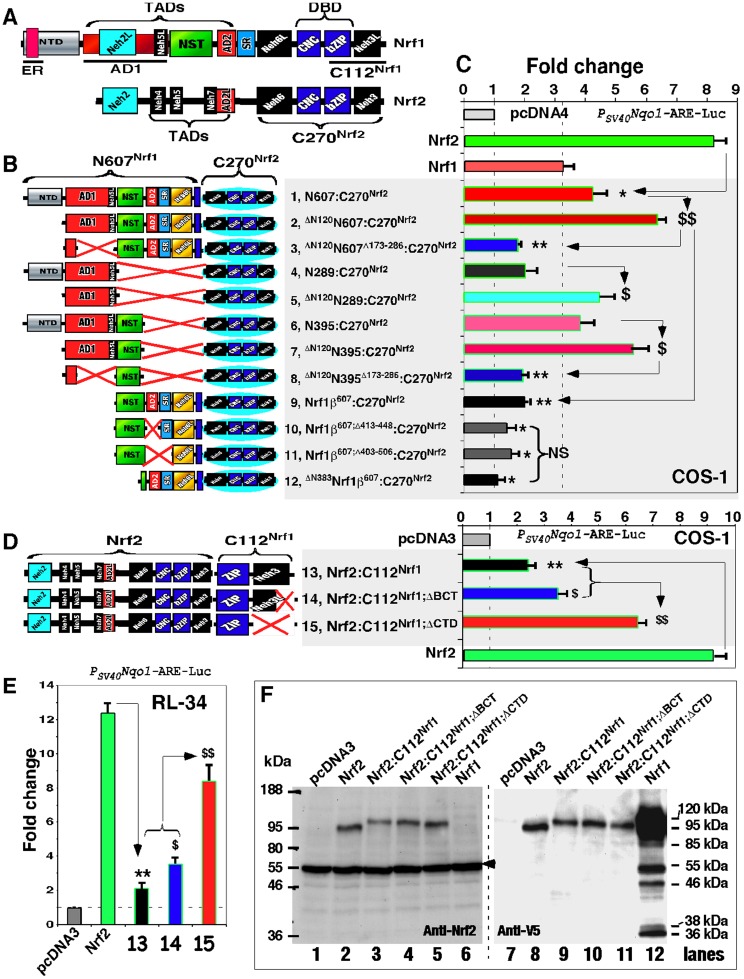
Nrf2-target gene expression is up-regulated by AD2 and NST domains of Nrf1 within chimaeras N607:C270^Nrf2^ and Nrf1β^N607^:C270^Nrf2^, but is also down-regulated by CTD of Nrf1 within additional chimaeras Nrf2:C112^ Nrf1^. (**A**) The *cartoon* shows structural domains of Nrf1 and Nrf2. The C-terminal residues 629–741 of Nrf1 (i.e. C112^Nrf1^) cover both its bZIP and CTD regions (*upper*). In Nrf2, the C270^Nrf2^ represents its C-terminal 270 aa between positions 328–597 that cover its Neh6, CNC, bZIP and Neh3 domains (*lower*). (**B**) Diagrammatic representation of chimaeras that were composed of various portions Nrf1 and Nrf2. The cDNA fragments encoding different portions of the N-terminal aa 1–607 of Nrf1 (e.g. N607^Nrf1^) and various lengths of the central aa 292–607 (i.e. Nrf1β^N607^) were ligated into the BamHI/EcoR1 sites of the Nrf2/pcDNA4His/Max B construct. Thus a series of chimaeric proteins were created by fusing different regions of either N607^Nrf1^ or Nrf1β^N607^ to the N-terminus of C270^Nrf2^. (**C**) The transactivation activity of the above-described chimaeric factors as well as wild-type Nrf1 and Nrf2. This was determined by using *P_SV40_Nqo1*-ARE-Luc and β-gal reporters that had been co-transfected with each of indicated expression constructs into COS-1 cells. The data are shown as fold changes (mean ± S.D.) of the transactivation activity when compared with the background (value of 1.0) that was measured from the blank co-transfection of cells with an empty pcDNA4 vector and the above two reporters. Thereafter, significant increases ($, p<0.05 and $$, p<0.001, n = 9) and decreases (*p<0.05, **p<0.001, n = 9) in activity relatively to the referenced activity are indicated (*arrows*). (**D**) Additional three chimaeras are schematically shown (*left panel*), which were created by fusing the full-length Nrf2 to the N-terminus of either C112^Nrf1^ or its mutants. These expression constructs for Nrf2 and its chimaeric proteins, together with *P_SV40_Nqo1*-ARE-Luc and β-gal reporters, were co-transfected into either COS-1 cells (*right panel* of ***D***) or RL-34 cells (**E**), before luciferase activity was assayed and the data are presented as fold changes (mean ± S.D). Significant increases ($, p<0.05 and $$, p<0.001, n = 9) and decreases (**p<0.001, n = 9) in activity were calculated relatively to the activity arrowed. (**F**) Total lysates of COS-1 cells that had been co-transfected with expression constructs for Nrf1, Nrf2 and its three chimaeric proteins (shown in ***D***) were resolved using 4–12% LDS/NuPAGE and then visualized by western blotting with antibodies against either Nrf2 or the V5 epitope (*left and right panels,* both blotting in the same gel-transferred nitrocellulose membranes). Amounts of protein loaded to each electrophoretic well were adjusted to ensure equal loading of β-gal activity. In addition, a non-specific protein band recognized by anti-Nrf2 antibody is arrowed (*right panel*).

Herein, whether AD2-containing TAD elements of Nrf1 make differential contributions to its chimaeric factors N607:C270^Nrf2^ and Nrf1β^607^:C270^Nrf2^ (Fig. 9A) are investigated by measuring *P_SV40_Nqo1-ARE-Luc* reporter gene assay. As shown in Figure 9B, N607:C270^Nrf2^ was created by fusing the N607 portion of Nrf1 with the C-terminal 270-aa portion of Nrf2 (designated C270^Nrf2^, which retains its Neh6, CNC, bZIP and Neh3 domains). Luciferase assay showed a ∼4.3-fold transactivation activity of N607:C270^Nrf2^ (Fig. 9C, *column 1*), which was modestly greater than the wild-type Nrf1 activity (i.e. ∼3.2-fold), but was much lesser than the wild-type Nrf2 activity (i.e. ∼8.2-fold). Removal of most of NTD from the N607 portion yielded the ^Δ120^N607:C270^Nrf2^ mutant, allowing its activity to be significantly increased to ∼6.5-fold (Fig. 9C, *columns 2*), while the transactivation activity was decreased to ∼1.6-fold by loss of most of AD1 (in the ^Δ120^N607^Δ173–286^:C270^Nrf2^ mutant) (Fig. 9C, *column 3*). These data indicate that N607:C270^Nrf2^ is positively regulated by the AD1 of Nrf1 and is negatively regulated by its NTD. By comparison with N607:C270^Nrf2^, the chimaeras N289:C270^Nrf2^ (i.e. the N289 portion contains AD1 and NTD) showed a lower activity (i.e. ∼2.0-fold), similar to that of ^Δ120^N607^Δ173–286^:C270^Nrf2^ (Fig. 9C, *columns 3 vs 4*), whilst the mutant chimaeras ^Δ120^N289:C270^Nrf2^ (in which AD1 of Nrf1 was retained with a loss of its NTD) exhibited a relatively higher activity (i.e. ∼4.4-fold), that was similar to that obtained from the entire N607:C270^Nrf2^ (Fig. 9C, *columns 5 vs 1*). Together, these data indicate that AD1-mediated transactivation is suppressed by NTD, whilst AD2 and the NST domain positively regulate reporter gene expression.

By comparison with N289:C270^Nrf2^, approximately 1.9- to 2.8-fold increases in the reporter gene activity were respectively observed in N395:C270^Nrf2^ (in which N395 contains its NTD, AD1 and NST domain) and its mutant ^Δ120^N395:C270^Nrf2^ (retaining AD1 and the NST domain) (Fig. 9C, *columns 6,7 vs 4*). Conversely, lack of most of AD1 (in the ^Δ120^N395 ^Δ173–286^:C270^Nrf2^ mutant) significantly decreased its transactivation activity to a ∼1.9-fold extent similar to those obtained from N289:C270^Nrf2^ or ^Δ120^N607^Δ173–286^:C270^Nrf2^ (Fig. 9C, *columns 8 vs 3, 4*). These results demonstrate that besides AD1, the NST domain of Nrf1 positively regulates N395:C270^Nrf2^.

Thereafter, we examined that both the NST domain and its adjoining AD2 contribute to the transcriptional activity of Nrf1β^607^:C270^Nrf2^ chimaeric factor (in which Nrf1β^607^ contains its NST, AD2, SR/PEST2 and Neh6L regions, see Fig. 9B). Luciferase assay showed that Nrf1β^607^:C270^Nrf2^ exerted a transactivation activity of ∼2.0-fold, that was similar to that of N289:C270^Nrf2^ (Fig. 9C, *columns 9 vs 4*). By contrast, the ^Δ383^Nrf1β^607^:C270^Nrf2^ mutant (lacking most of the NST domain) showed a decreased activity (Fig. 9C, *column 12*), which is similar to the background value measured from COS-1 cells that had been transfected with an empty pcDNA4 vector. Both Nrf1β^607;Δ413–448^:C270^Nrf2^ (lacking the major acidic-amphipathic portion of AD2) and Nrf1β^607;Δ403–506^:C270^Nrf2^ (lacking both the entire AD2 and SR/PEST2 regions) showed similar reduction of ∼1.4-fold activity (Fig. 9C, *columns 10 *&* 11*). These findings indicate that Nrf1β^607^:C270^Nrf2^ is positively regulated by AD2 and the NST domain of LCR-F1/Nrf1β, but is negatively regulated by its SR/PEST2 or Neh6L regions.

### Inhibition of Nrf2-target gene expression by attachment of the CTD to Nrf2

To explore whether attachment of the CTD from Nrf1 to Nrf2 exert a negative effect on target gene expression, we created a chimaeric factor Nrf2:C112^Nrf1^ by fusing the C-terminal 112-aa portion of Nrf1 (designated C112^Nrf1^, that contains its ZIP and CTD) to the C-terminus of wild-type Nrf2. Luciferase assays of COS-1 and RL34 cells showed that the chimaeras Nrf2:C112^Nrf1^ exerted a ∼2.2-fold activity, that was much lower than the wild-type Nrf2 (Fig. 9, D and E, *column* 13), implying that attachment of C112^Nrf1^ to Nrf2 suppresses its target gene activity. Conversely, removal of the CTD from the C112^ Nrf1^ portion (to yield the Nrf2:C112^Nrf1;ΔCTD^ mutant) resulted in the major recovery of Nrf2-target reporter gene to be re-activated by ∼6.5-fold (in COS-1 cells) or by ∼8.5-fold (in RL-34 cells), indicating that, within the mutant chimaeras, the Nrf2 portion is liberated from the suppression by attached CTD from Nrf1 (Fig. 9, D and E, *column* 15). Removal of BCT also caused a modest increase in the reporter gene transactivation mediated by Nrf2:C112^Nrf1;ΔBCT^ (*column* 14), suggesting partial inhibition of Nrf2:C112^Nrf1^ by the basic c-tail ^730^RRQERKPKDRRK^741^ of Nrf1. The difference between these chimaeric activities is not associated with their protein amounts, because no changes in the abundance of these chimaeric proteins were observed by western blotting of COS-1 cells (Fig. 9F). Overall, these data indicate that the chimaeric Nrf2:C112^Nrf1^ factor is endowed with the CTD within the C112^Nrf1^ portion to down-regulate Nrf2-target gene expression.

### Endogenous ARE-driven genes are up-regulated by Nrf1 and LCR-F1/Nrf1β whilst down-regulated by Nrf1γ and Nrf1δ

**Figure 10:**
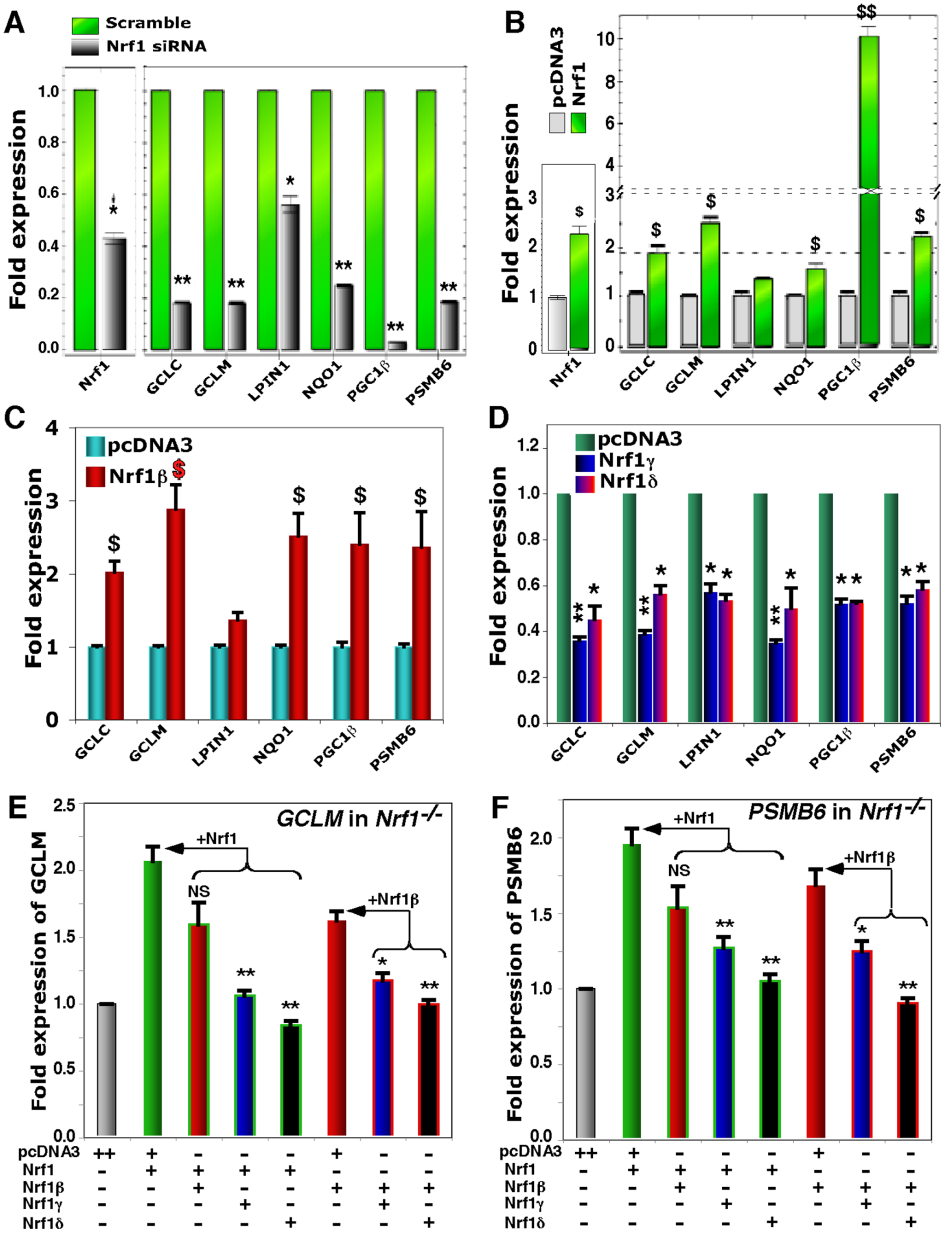
Endogenous genes are up-regulated by Nrf1 and Nrf1β/LCR-F1 but also down-regulated by Nrf1γ and Nrf1δ. (**A**) Knockdown of Nrf1 by its targeting siRNA, which, along with a scramble siRNA (as an internal control), was transfected into HEK 293T cells as described previously [12] (and maintained in our laboratory). Subsequently, changes in the mRNA expression of both the endogenous *Nrf1 per se* and Nrf1-target genes were analyzed by real-time qPCR. The data are shown as fold changes (mean ± S.D) in gene knockdown by Nrf1-siRNA relatively compared to the scramble value (1.0 set). Significant decreases (*p<0.005, **p<0.001, n = 9) in gene expression relatively to the basal level are indicated. (**B** to **D**) Expression constructs for Nrf1 (***B***), Nrf1β (***C***), Nrf1γ and Nrf1δ (***D***) (2 µg of cDNA each, along with an empty pcDNA3 control vector) were transfected into HEK 293T cells. Thereafter, alterations in the expression of Nrf1-target genes were determined by real-time qPCR, and were calculated as fold changes (mean ± S.D) in gene regulation by distinct Nrf1 isoforms when compared to the background (value of 1.0). Significant increases ($, p<0.05 and $$, p<0.001, n = 9) and decreases (*p<0.005, **p<0.001, n = 9) in gene expression relatively to the basal level are indicated. (**E** and **F**) Nrf1 and Nrf1β, Nrf1γ and Nrf1δ were restored into *Nrf1^−/−^* MEFs, in which Nrf1 has been lost (see Fig. S3) before being transfected with expression constructs for distinct isoforms alone or in combination, which were indicated (+, 1 µg of cDNA; ++, 2 µg of cDNA). Subsequently, real-time qPCR was performed to determine changes in the expression of *GCLM* (***E***) and *PSMB6* (***F***). The data are presented as folds (mean ± S.D) relatively to the blank transfection with pcDNA3 alone (value of 1.0). Significant decreases (*p<0.005, **p<0.001, n = 9) in gene expression were calculated when compared to the level of genes regulated by Nrf1 or Nrf1β (*arrows*).

Several previous studies [17], [21], [22], [54], [55] have showed that Nrf1 regulate the constitutive expression of a subset of ARE-driven genes responsible for maintaining cellular redox protein and lipid homeostasis. Real-time qPCR analysis of HEK-293T cells that had been transfected with *Nrf1*-targeting siRNA revealed that knockdown of this factor resulted in a significant decrease in the endogenous expression of mRNAs of *GCLC* (glutamate-cysterne ligase catalytic subunit), *GCLM* (glutamate-cysteine ligase catalytic modifier subunit), *NQO1* (NAD(P)H:quinone oxidoreductase 1) and *PSMB6* (the 26S proteasomal subunit B6) to ∼20–25% as compared to their basal levels measured from the cells transfected with a scrambled control siRNA (Fig. 10A).

Recently, it has been showed that Nrf1 is essential for the hepatic lipid homeostasis through regulating both transcriptional coactivator genes *Lipin1* (LPIN1, also identified as a phosphatidate phosphatase) and *PGC-1β* (peroxisome proliferator-activated receptor-γ (PPAR-γ) coactivator-1β) [56]. As expected, our data showed that expression of *PGC-1β* was almost completely abolished upon siRNA knockdown of Nrf1 to ∼42% of its basal level, accompanied with a similar reduction in expression of *Lipin1* (Fig. 10A).

Over-expression of the full-length Nrf1 that had been transfected in HEK-293T cells (Fig. 10B) caused a ∼1.5–2.5-fold increase in expression of endogenous *GCLC*, *GCLM, NQO1* and *PSMB6*. The parallel experiments also showed that expression of *PGC-1β* was markedly up-regulated to ∼10-fold by Nrf1, whilst no significant change in expression of *Lipin1* was observed. More interestingly, similar expression patterns of such genes, with an exception of *PGC-1β* which was only induced by ∼2.5-fold, as determined in HEK-293T cells that had been transfected with LCR-F1/Nrf1β (Fig. 10C). However, over-expression of either Nrf1γ or Nrf1δ resulted in a significant decrease in expression of the genes examined (Fig. 10D).

Collectively, the above results demonstrate that differential expression of endogenous ARE-driven genes is up-regulated by Nrf1 and LCR-F1/Nrf1β, but is down-regulated by Nrf1γ and Nrf1δ. Differential contributions of these distinct isoforms to gene regulation were further determined by restoring ectopic Nrf1, LCR-F1/Nrf1β, Nrf1γ or Nrf1δ into *Nrf1^−/−^* MEFs (in which aa 296–741 of endogenous Nrf1 had been genetically deleted), followed by real-time qPCR analysis of the resulting expression of *GCLM* (Fig. 10E) and *PSMB6* (Fig. 10F). When compared with the basal *GCLM* and *PSMB6* levels measured from transfection of *Nrf1^−/−^* MEFs with an empty pcDNA3 vector, their transcriptional expression was increased by restoration of Nrf1 and Nrf1β; the increased expression of *GCLM* and *PSMB6* mediated by Nrf1 and Nrf1β was significantly prevented by Nrf1γ or Nrf1δ (Fig. 10, E and F), though no significant difference between the overall abundances of restored Nrf1 isoforms was measured (Fig. S3). Intriguingly, co-transfection experiments showed that Nrf1-mediated transcriptional expression of *GCLM* and *PSMB6* seemed to be modestly blunted by the relatively weak Nrf1β to a similar extent to that of Nrf1β restored alone (Fig. 10, E and F). Together with the data (as shown in Fig. 7B) and our previous results [12], [48], these findings give rise to a possibility that the nuclear-localized LCR-F1/Nrf1β (i.e. 55-kDa) and its further degraded polypeptides (between 38-kDa and 25-kDa, acting as dominant-negative regulators) may gain more access to endogenous genes than the membrane-associated Nrf1 protein such that they occupationally bind target genes competitively against the intact Nrf1 factor.

## Discussion

In the present study we found that: (i) both Nrf1 and its shorter isoform LCR-F1/Nrf1β are negatively regulated by their CTD and Neh6L regions, but both are positively regulated by their AD2 and NST domains; however a dual opposing effect of SR/PEST2 on Nrf1 is not exerted on LCR-F1/Nrf1β; (ii) the latter LCR-F1/Nrf1β is a relatively weak activator to the full-length Nrf1 factor, whilst the small molecular-weight Nrf1γ and Nrf1δ act as two dominant-negative forms that competitively inhibit the wild-type Nrf1 and Nrf2, as well as LCR-F1/Nrf1β; and (iii) differential expression of endogenous ARE-driven genes is up-regulated by Nrf1 and LCR-F1/Nrf1β, besides Nrf2, but is down-regulated by Nrf1γ and Nrf1δ. These together control the overall activity of Nrf1 to fine-tune the steady-state expression of target genes for maintaining cellular homeostasis.

### LCR-F1/Nrf1β is a weak activator relative to the full-length Nrf1; both are inhibited by the small dominant-negative Nrf1γ and Nrf1δ

Accumulating evidence reveals that over eleven Nrf1 isoforms [46] are produced from the single *nfe2l1* gene, though they are differentially expressed in different mammalian species [1], [57]–[60]. Upon translation, the mouse Nrf1 is biosynthesized as an unglycosylated 95-kDa polypeptide. During the membrane-topogenic vectorial process, the nascent 95-kDa polypeptide is co-translationally integrated within the ER, where it is glycosylated to become a 120-kDa glycoprotein [45], [46]. Subsequently, the 120-kDa glycoprotein is partially repartitioned out of ER into the cyto/nucleoplasmic compartments, whereupon Nrf1 is deglycosylated to generate an active 95-kDa transcription factor [45]. Thereafter, the membrane-bound 95-kDa Nrf1 proteins are subjected to selective proteolytic processing to yield multiple cleaved polypeptides of between 85-kDa and 25-kDa [48]. In addition to the post-translational processing of Nrf1, its isoforms ranging between 65-kDa and 25-kDa can also be synthesized by translation through distinct in-frame start ATG codons embedded in various lengths of mRNA sequences, some of which arise from alternative splicing of variant transcript species [9], [49], [50], [57], [58], [61].

Amongst these Nrf1 isoforms, its polypeptides of ∼55-kDa, 36-kDa and 25-kDa are originally designated LCR-F1/Nrf1β [12], [59], [61], Nrf1γ and Nrf1δ [46], [59], respectively. LCR-F1/Nrf1β migrates at a mass of 55-kDa in the pH 7.0 LDS/NuPAGE gel ([12], [44] and herein), but the pH 8.9 Laemmli SDS-PAGE allows its mobility to be exhibited at an estimated size of 60-kDa [19] or 65-kDa (called p65Nrf1 [62]). To provide a clear explanation of the Nrf1 nomenclature in the literature, we also note a recent report on another confused Nrf1β [63]. In fact, this spliced variant was originally designated as Nrf1 clone Δ767 in 1998 [57] (GenBank accession No. NM_001130453.1); it was translated as an N-terminally-truncated mutant lacking 181-aa of Nrf1 (spanning the entire NTD and one-third of the AD1) and thus was simply designated Nrf1^ΔN^ in 2009 [46], [59], although additional 12 aa (i.e. MGWESRLTAASA) replaced the original 181-aa of Nrf1.

By comparison with the full-length Nrf1, the 55-kDa Nrf1β/LCR-F1 lacks both the NTD and the essential transactivation domain AD1, but still retains AD2 and NST domain required for its transactivation activity. It is thus postulated that Nrf1β/LCR-F1 is a nuclear activator with a poor transactivation activity; this has been confirmed by us and others [12], [57], [58], [61]. Herein, our experiments confirm that endogenous Nrf1-target genes and ARE-driven luciferase reporters are up-regulated by LCR-F1/Nrf1β, as it acts as a *bona fide* activator with a weak activity relative to the wild-type Nrf1. On the other side, co-transfection of LCR-F1/Nrf1β- and Nrf1-expressed constructs appears to slightly decrease Nrf1-mediated transactivation of endogenous genes, but not ectopic reporter genes, to a similar extent to that of transactivation mediated by LCR-F1/Nrf1β alone. Together with previous publications [12], [48], [60], [61], [64], these findings indicate that the ER membrane-bound Nrf1 has tempo-spatial behaviour, that is distinct from the nuclear water-soluble LCR-F1/Nrf1β, to bind ARE sequences in Nrf1-target genes within different transcriptional assembly being built in different cell types. However, an exception was reported that LCR-F1/Nrf1β was thought to be a significant dominant-negative inhibitor of ARE-driven gene transactivation against the wild-type Nrf1 and Nrf2 [62]. The dispute on LCR-F1/Nrf1β suggests that it is an unstable protein that might be proteolytically processed to yield several small poplypeptides of 36-kDa and 25-kDa; this is supported by the fact that the activity of Nrf1β/LCR-F1 is significantly increased by blocking generation of either 36-kDa Nrf1γ or 25-kDa Nrf1δ polypeptides. Induction of Nrf1β/LCR-F1 activity may also be dependent on distinct stressors in different cell lines [57], [58], [64].

By definition, a dominant-negative mutant usually disrupts certain functions of the intact protein (i.e. *negative*) and also out-compete the endogenous protein in some way (i.e. *dominant*) [65], [66], it is deduced that the 36-kDa Nrf1γ and the 25-kDa Nrf1δ, but not the 55-kDa Nrf1β/LCR-F1, act as *bona fide* dominant-negative isoforms. This is supported by lack of all the potential transactivation elements (i.e. AD1, AD2 and NST domain) in Nrf1γ and Nrf1δ [12], [57], [67]. Our evidence presented demonstrates that over-expression of either Nrf1γ or Nrf1δ almost abolishes transcriptional expression of endogenous ARE-battery genes and ectopic luciferase reporters against the wild-type Nrf1 or Nrf2. Further co-transfection experiments confirm that both Nrf1γ and Nrf1δ dominant-negatively inhibit the transactivation activity of Nrf1 and Nrf2. This occurs possibly through competitive interference with the intact functional assembly of the active transcription factor complex that binds ARE sequences in target genes.

### Both Nrf1 and LCR-F1/Nrf1β are positively regulated by their AD2 and NST domains but also are negatively regulated by their CTD and Neh6L regions

Our previous work showed that the full-length Nrf1 is a modular protein containing nine discrete domains, and both its NTD and AD1 are lost in the short LCR-F1/Nrf1β [43], [45]. Besides AD1, both AD2 and NST domain also contribute to the full activity of Nrf1 [12], [59]. In addition, the SR domain (aa 455–488) was early considered to function as a transactivation domain in the human Nrf1 and its long form TCF11 [68]. We recently found that the core SR region (aa 466–488) contributes to the basal and stimulated activity of Nrf1 [45]. However, removal of aa 403–506 covering the entire AD2 and most of SR/PEST2 causes a marked augmentation in the basal and stimulated activity of Nrf1 to a similar extent to that obtained from deletion of aa 413–448 covering a short core AD2 sequence [45]. In combination with our recent findings [45], [48], these data indicate that both AD2 and SR also encompass negative regulatory degrons to inhibit the full-length Nrf1; this effect depends on distinct membrane-topological conformation (that enables repositioning of their adjoining degrons to target the protein for proteolytic degradation). However, removal of aa 403–506 from the water-soluble LCR-F1/Nrf1β does not cause a further decrease in its activity, when compared to deletion of aa 413–448 (in this study), suggesting that AD2, but not the SR domain, is required for its transactivation activity. Moreover, the Gal4-based reporter experiments demonstrate that AD2 and NST rather than SR domains contribute to transactivation mediated by LCR-F1/Nrf1β; this notion is supported by evidence obtained from the chimaeric N607^Nrf1^:C270^Nrrf2^ factor.

Previous studies have reported that Nrf1 is negatively regulated by its NTD and Neh6L regions from within the molecule through different mechanisms [43], [48]. Our present work uncovers that beside NTD and Neh6L, CTD of Nrf1 also contributes to the negative regulation of both the full-length CNC-bZIP factor and its shorter isoform Nrf1β/LCR-F1, whilst the dominant-negative effect of Nrf1γ or Nrf1δ is unaffected by its CTD. The inhibitory effect of CTD is also elicited within its chimaeric factor resulting from attachment of CTD to Nrf2 that enables its transactivation activity to be diminished. However, the detailed mechanism(s) underlying the negative regulation by CTD of Nrf1, particularly Nrf1β/LCR-F1, remains to be determined. During topogenesis of the full-length Nrf1, topological folding of its TMc-containing CTD around membranes is dictated by the N-terminal topogon [12], [46], and thus removal of CTD could enables the CNC-bZIP protein to be dislocated to the cyto/nucleoplasmic side of the ER membrane such that CTD-deficient mutant would gain more access to Nrf1-target genes than the full-length protein, thereby increasing its activity. Huang and colleagues reported that the CTD and its adjacent bZIP domain enable LCR-F1/Nrf1β to directly interact with either the cytomegalovirus immediate-early protein 2 (IE2) [69] or a cell cycle-dependent microspherule protein 2 (MCRS2, that is involved in telomere shortening by inhibiting telomerase activity [70]). As a consequence, the transactivation activity of LCR-F1/Nrf1β is repressed by IE2 and MCRS2, although both its heterodimerization with a small Maf protein and its ability to bind ARE sequences in Nrf1-target genes are unaffected by these two interacting partners.

Our previous studies showed that LCR-F1/Nrf1β and Nrf1γ are primarily located in the nucleus, but another small portion of these two proteins are recovered in the membrane fraction, that are not protected by membranes and hence are rapidly digested by proteases in membrane protection assays [12], [45], [48]. In this study, confocal microscopy revealed that the basic c-tail ^730^RRQERKPKDRRK^741^ does not appear to act as a functional nuclear localization signal, because removal of the basic peptide causes the resultant mutant protein to be modestly accumulated in the nucleus of some cells. Live-cell imaging experiments unravel that most of the extra-nuclear proteins of GFP-CTD or its mutants lacking various lengths of aa 714–741 from CTD are rapidly digested by PK in around 10–20 min. These indicate that the extra-nuclear fraction of GFP-CTD fusion protein could be associated with the cytoplasmic leaflet of membrane possibly through amphipathic or electrical interactions. In addition, attachment of CRAC5 from CTD to the C-terminus of GFP (to yields GFP-CTD^Δ701–741^) allowed the fusion protein to be insensitive to PK digestion, relative to GFP-CTD. Collectively, these findings suggest that CRAC5, along with the amphipathic TMc region, enables the protein to be associated with the cholesterol-rich membrane microdomain (e.g. rafts). Together with the fact that CRAC5 is present in the Neh3L-containing CTD of Nrf1 but is absent from the Neh3 domain of Nrf2, therefore we envisage that the motif is attributed to the difference of Neh3L from Neh3.

## Concluding Comments

Collectively, Nrf1, LCR-F1/Nrf1β and Nrf1γ are three major representatives of various isoforms arising from both post-transcriptional and/or post-translational processing of the *nfe2l1* gene products. Together with previous publications [12], [57], [58], [61], [64], the evidence that has been provided herein demonstrates that Nrf1β/LCR-F1 is *per se* a weak activator relative to the intact full-length Nrf1, and that the shorter isofrom does not functions as a *de facto* dominant-negative inhibitor of Nrf1 (and Nrf2), albeit it can modestly blunt wild-type Nrf1 activity to mediate endogenous ARE-driven gene expression. By contrast, Nrf1γ and Nrf1δ act as *bona fide* dominant-negative isoforms that competitively inhibit Nrf1 and LCR-F1/Nrf1β. We have also presented further evidence showing that both Nrf1 and LCR-F1/Nrf1β are positively regulated by their AD2 and NST domains, but also are negatively regulated by their Neh6L and CTD regions. However, CTD does not alter the dominant-negative effect of Nrf1γ. Intriguingly, it is to note that CTD of Nrf1 is homologous to the equivalent Neh3 domain of Nrf2; the latter Nrf2 is positively regulated by its Neh3 domain through direct interaction with CHD6 [37], but CHD6 is not detected as one of Nrf1-interacting proteins by the liquid chromatography-mass spectrometry (LC-MS)/MS [71]. Our previous studies indicated that the negative regulation of Nrf1 by CTD is associated with its topological folding within and around membranes [12], [46], and the association could be enhanced by CRAC5 through possible interaction with the cholesterol-rich microdomain. Moreover, LCR-F1/Nrf1β is repressed by IE2 [69] and MCRS2 [70] through interaction with its CTD-adjoining region, whilst both its heterodimerization with small Maf protein and its activity of DNA-binding to target genes are unaffected. However, the detailed mechanism(s) underlying negative regulation of Nrf1 by its Neh3L-containing CTD remains to be deeply studied in order to elucidate distinction between Neh3L (particularly in LCR-F1/Nrf1β and Nrf1^ΔN^) and Neh3 of Nrf2.

## Supporting Information

Figure S1 Alignment of amino acids covering the CTD of Nrf1 from 30 different species. The CTD of Nrf1 is highly conserved amongst different vertebrate species, particularly mammalian animals. This domain is composed of CRAC5, PYxP, TMc and Basic C-tail, and its secondary structure is predicated and shown in the main text.
(TIF)

Figure S2 Live-cell imaging of GFP-CTD^Δ701 −741^ retaining CRAC5. COS-1 cells co-expressing GFP-CTD^Δ701 −741^ (that lacks most of the CTD of Nrf1, but retains its CRAC5 only), together with the ER/DsRed marker, were subjected to live-cell imaging combined with the *in vivo* membrane protease protection assay. The cells were first permeabilized by 20-µg/ml digitonin for 10 min, before being co-incubated with 50-µg/ml PK for 90 min. The imaging data are shown that were obtained from 90 min digestion by PK (**A**), and the images obtained from 30 min to 48 min incubation with PK are presented (**B**).
(TIF)

Figure S3 Restoration of distinct Nrf1 isoforms in *Nrf1^−/−^* cells. *Nrf1^−/−^* MEFs were allowed for restored expression of distinct Nrf1 isoforms followed by real-time qPCR analysis of Nrf1-target gene expression. The results showed no significant difference between the mRNA levels of Nrf1 expressed in distinct transfected cells.
(TIF)
