# Supplementary figures and images for: The C-Terminal Domain of Nrf1 Negatively Regulates the Full-Length CNC-bZIP Factor and Its Shorter Isoform LCR-F1/Nrf1β; Both Are Also Inhibited by the Small Dominant-Negative Nrf1γ/δ Isoforms that Down-Regulate ARE-Battery Gene Expression
Source: PLoS One. 2014 Oct 7;9(10):e109159. doi: 10.1371/journal.pone.0109159 (PMC4188613; doi:10.1371/journal.pone.0109159)

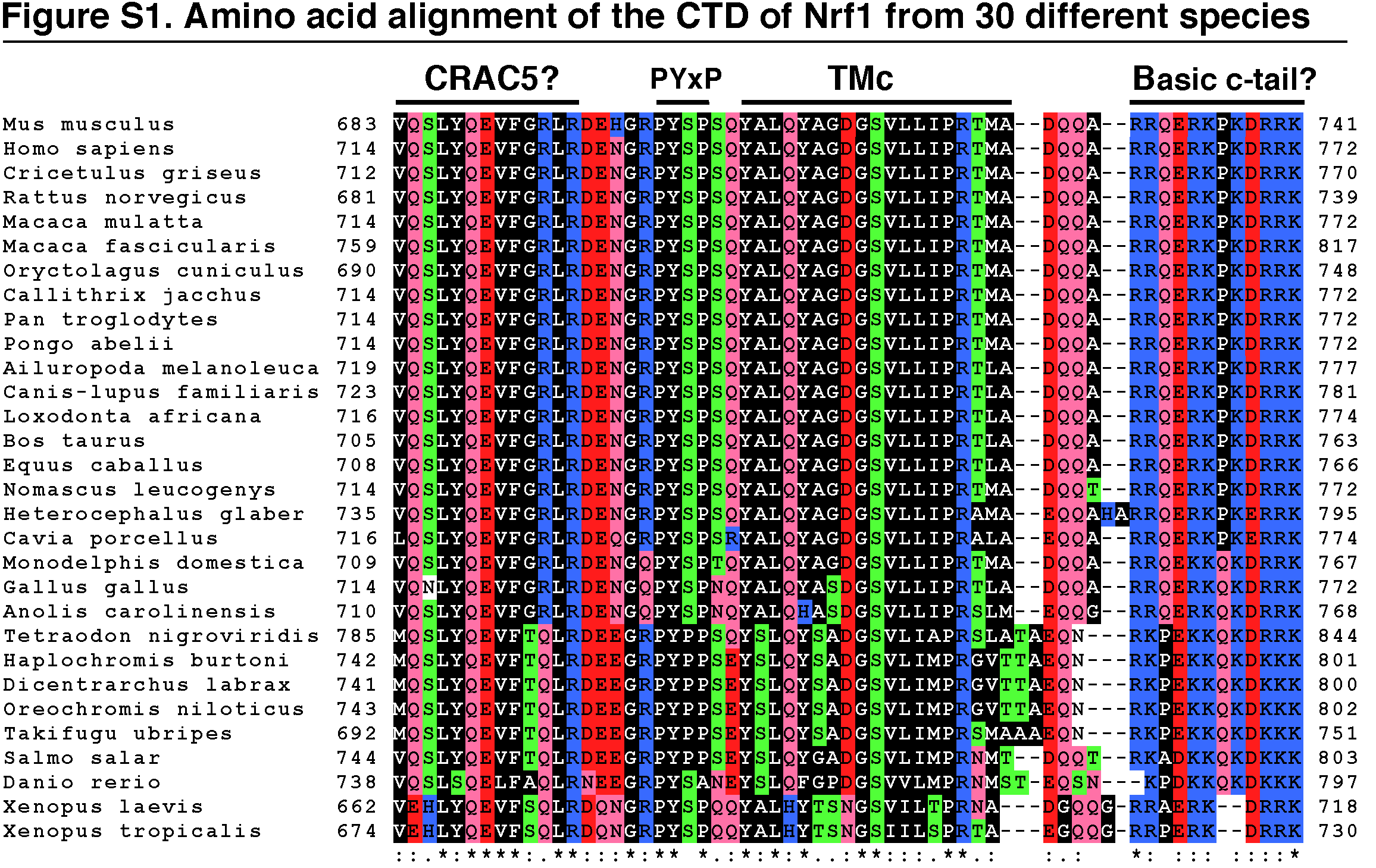

Supplement: Figure S1 — Alignment of amino acids covering the CTD of Nrf1 from 30 different species. The CTD of Nrf1 is highly conserved amongst different vertebrate species, particularly mammalian animals. This domain is composed of CRAC5, PYxP, TMc and Basic C-tail, and its secondary structure is predicated and shown in the main text. (TIF) [file pone.0109159.s001.tif]

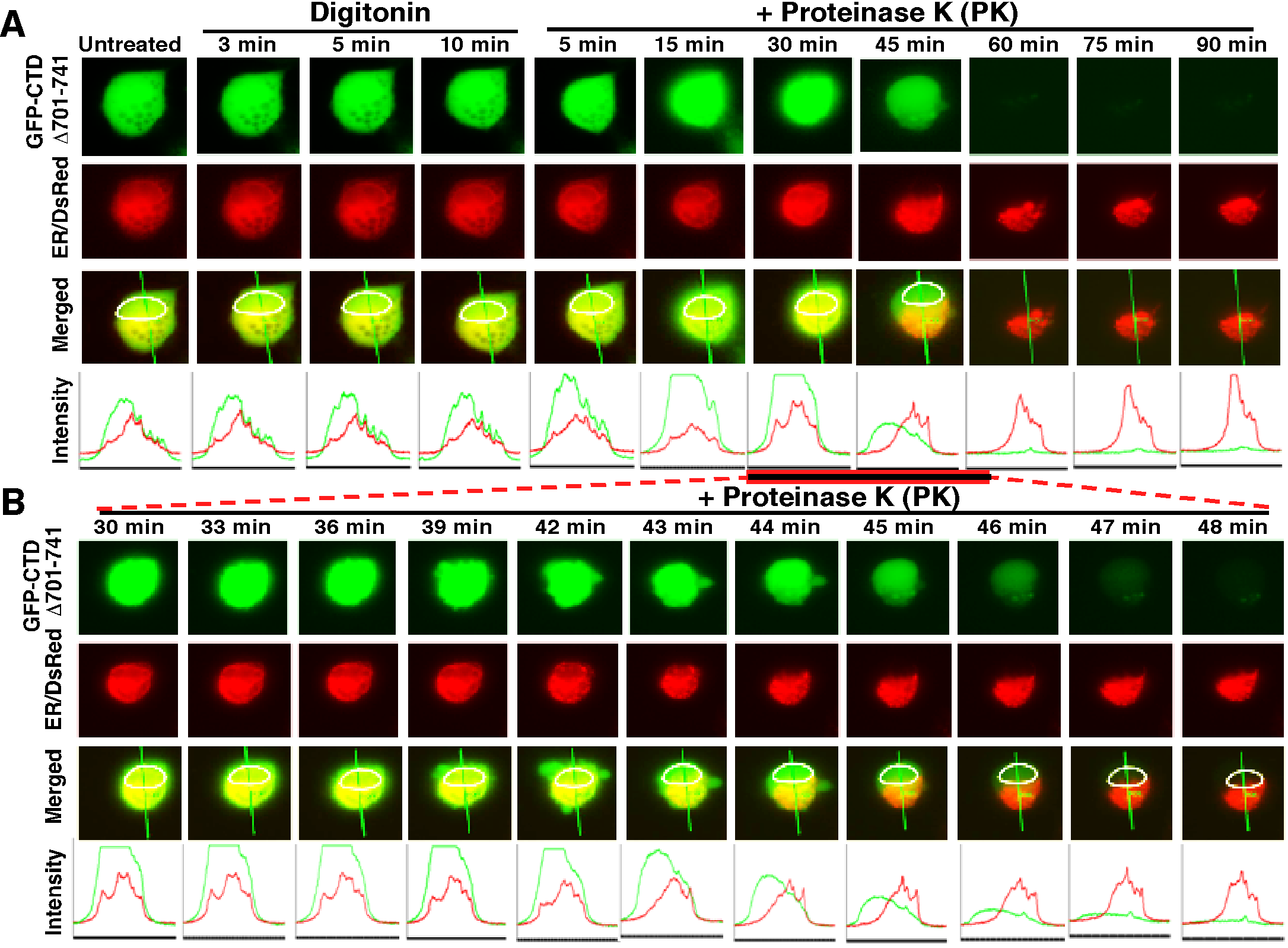

Supplement: Figure S2 — Live-cell imaging of GFP-CTDΔ701 −741 retaining CRAC5. COS-1 cells co-expressing GFP-CTDΔ701 −741 (that lacks most of the CTD of Nrf1, but retains its CRAC5 only), together with the ER/DsRed marker, were subjected to live-cell imaging combined with the in vivo membrane protease protection assay. The cells were first permeabilized by 20-µg/ml digitonin for 10 min, before being co-incubated with 50-µg/ml PK for 90 min. The imaging data are shown that were obtained from 90 min digestion by PK (A), and the images obtained from 30 min to 48 min incubation with PK are presented (B). (TIF) [file pone.0109159.s002.tif]

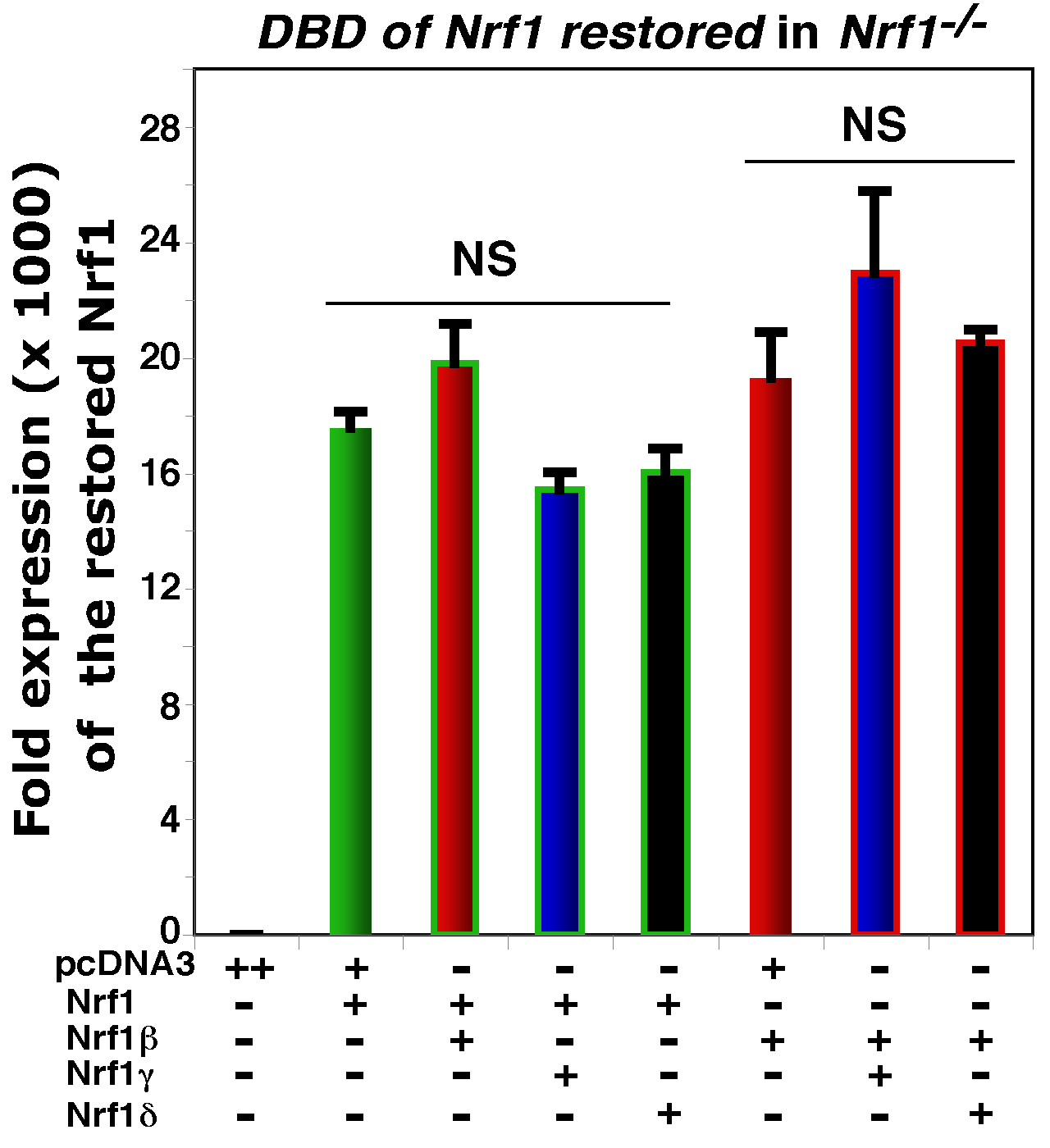

Supplement: Figure S3 — Restoration of distinct Nrf1 isoforms in Nrf1−/− cells. Nrf1−/− MEFs were allowed for restored expression of distinct Nrf1 isoforms followed by real-time qPCR analysis of Nrf1-target gene expression. The results showed no significant difference between the mRNA levels of Nrf1 expressed in distinct transfected cells. (TIF) [file pone.0109159.s003.tif]
